# The potential of plant-derived triterpenoids as biological nitrification inhibitors

**DOI:** 10.1007/s00253-026-13776-3

**Published:** 2026-03-17

**Authors:** Hugo Ribeiro, Evangelia S. Papadopoulou, Kunyang Zhang, Alexandros E. Kanellopoulos, Kalliope K. Papadopoulou, Dimitrios G. Karpouzas

**Affiliations:** 1 Laboratory of Plant and Environmental Biotechnology, Department of Biochemistry and Biotechnology, University of Thessaly, Larissa, Greece; 2 Laboratory of Environmental Microbiology and Virology, Department of Environmental Sciences, University of Thessaly, Larissa, Greece; 3 Department of Environmental Chemistry, Überlandstrasse 133, 8600 Dübendorf, Switzerland; 4 Department of Chemistry, University of Zürich, Winterthurerstrasse 190, 8057 Zurich, Switzerland

**Keywords:** Plant-derived triterpenoids, Biological nitrification inhibitors (BNIs), Ammonia-oxidizing archaea (AOA), Ammonia-oxidizing bacteria (AOB), Sustainable agriculture

## Abstract

**Abstract:**

Biological nitrification inhibitors (BNIs) present an environmentally friendly approach to reduce nitrogen losses and enhance nitrogen use efficiency, with plant-derived triterpenoids emerging as promising candidates. We evaluated 18 triterpenoids as BNIs using *in vitro* assays with soil ammonia-oxidizing bacteria (AOB) (*Nitrosospira multiformis*, *Nitrosomonas ureae*) and archaea (AOA) (*Nitrososphaera viennensis*, *Nitrosotalea sinensis*) at high and low concentrations. A Graph Neural Network framework was applied to predict nitrification inhibition (NI) and identify structural features, including key functional groups, linked to inhibitory patterns. Triterpenoids were more active on AOA, demonstrating higher efficacy than sakuranetin (a known BNI), but did not inhibit AOB. Six triterpenoids showed inhibitory activity on AOA (29–100%), with 3-O-acetyl-11-keto-beta boswellic acid and 11-keto-beta boswellic acid as the most potent inhibitors (ammonia oxidation inhibition > 94%), followed by echinocystic acid (> 87%), ursolic acid (> 74%), asiatic acid (> 65%), and echinocystic acid-3-O-glucoside (29–94%). *In silico* analyses predicted accurately the activity of model inhibitors such as DMPP, MHPP, and ethoxyquin on AOB and AOA, respectively, and the limited activity of triterpenoids on AOB, but did not predict their strong inhibitory effects on AOA, underscoring the need for expanded datasets for model refinement. The selective activity of some triterpenoids on AOA is hypothesized to involve interference with 3-hydroxy-3-methylglutaryl-CoA reductase, a key enzyme in archaeal membrane biosynthesis, although this requires experimental validation. Still, strain-specific responses suggest the involvement of additional mechanisms. This study provides the first experimental evidence for the potential of plant-derived triterpenoids as BNIs, supporting their relevance for sustainable agriculture.

**Graphical Abstract:**

**Figure:**
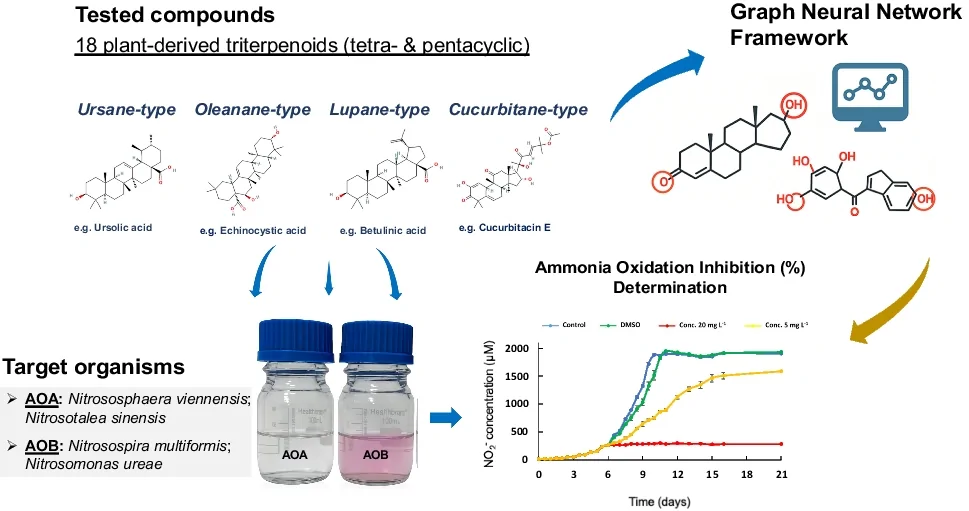

**Key points:**

• *Triterpenoids strongly inhibited AOA but had no effect on AOB nitrification activity.*

• *Six ursane/oleanane-type triterpenoids showed strong AOA inhibition beyond known BNIs.*

• *Inhibition patterns suggest triterpenoid structure relates to AOA selectivity.*

**Supplementary Information:**

The online version contains supplementary material available at 10.1007/s00253-026-13776-3.

## Introduction

Nitrogen (N) is an essential nutrient for plant growth and a key component of fertilizers that enhances crop yields and food production (Spiertz 2009). Its availability in soil directly affects the productivity of agroecosystems, making it a vital element in modern agriculture (Govindasamy et al. 2023). However, the application of N-fertilizers often exceeds crop needs, leading to undesirable N losses through various processes such as leaching, volatilization, and denitrification (Tei et al. 2020). These processes release reactive nitrogen into the environment, mainly as nitrate (ΝΟ_3_^−^) and nitrous oxide (N_2_O)—a potent greenhouse gas with a 100-year global warming potential 273 times higher than that of CO_2_ (Tian et al. 2020)—thereby reducing fertilizer efficiency and causing environmental degradation, including nitrate contamination of water bodies, greenhouse gas emissions, and soil acidification (Martínez-Dalmau et al. 2021). This imbalance leads to low nitrogen use efficiency (NUE) in agricultural systems and contributes to the disruption of the global Ν cycle, with agricultural N inputs now exceeding the safe planetary boundaries for nitrogen (Schulte-Uebbing et al. 2022). Increasing NUE is therefore crucial for achieving sustainable agriculture within the safe operating space for nitrogen. A number of management strategies have been used to improve NUE in agricultural soils (Hutchings et al. 2020) with particular focus on decelerating nitrification, the microbial conversion of ammonium to nitrate, as a key process controlling N losses (Klimasmith and Kent 2022). Application of synthetic nitrification inhibitors (SNIs), such as nitrapyrin, dicyandiamide (DCD), and 3,4-dimethylpyrazole phosphate (DMPP) as mixtures with N fertilizers have been the main approach to decelerate nitrification and enhance NUE (Beeckman et al. 2024). In general, SNIs have been postulated to inhibit ammonia monooxygenase (AMO), an enzyme controlling the first step of nitrification, the transformation of ammonia to hydroxylamine, which is shared by ammonia-oxidizing archaea (AOA), ammonia-oxidizing bacteria (AOB), and comammox bacteria (Ruser and Schulz 2015). However, SNIs have been criticized for erratic performance across different soil types (Papadopoulou et al. 2022), high costs (Qiao et al. 2015), and potential environmental safety concerns (Woodward et al. 2016; Kösler et al. 2019). Due to these limitations and the increasing demand for more natural solutions, biological nitrification inhibitors (BNIs) have gained attention as a potential alternative.

Typically, BNIs are secondary metabolites produced by certain plants as root exudates or isolated from root tissue and considered an adaptive mechanism that allows plants to conserve nitrogen in N-limited ecosystems (Subbarao et al. 2006; Qiao et al. 2015; Ma et al. 2021). To date, several plant species, primarily grasses, have been identified as capable of exuding or producing BNIs. Examples of such molecules are methyl 3-(4-hydroxyphenyl)propionate (MHPP), sorgoleone, and sakuranetin exuded by sorghum (*Sorghum bicolor*) roots; 1,9-decanediol and syringic acid produced by rice (*Oryza sativa*); and 2,7-dimethoxy-1,4-naphthoquinone (zeanone) and 6-methoxy-2(3H)-benzoxazolone (MBOA) from maize (*Zea mays*) (Nardi et al. 2020; Subbarao et al. 2009, 2013; Zakir et al. 2008; Lu et al. 2022; Otaka et al. 2022, 2023). Research on BNIs is still in its infancy, and other non-tested plant-derived secondary metabolites may also exhibit BNI activity.

Plant secondary metabolites can be categorized into three main chemical groups: phenolics (including tannins), terpenes, and alkaloids (Hilal et al. 2024). Terpenes represent the largest and most diverse group; although their biosynthesis follows a common pathway based on the fusion of C5 units with an isoprenoid structure (C_5_H_8_), they exhibit remarkable structural diversity (Tholl 2015). Triterpenes constitute a large class of natural products, comprising well over 100 distinct skeletons, including sterols, steroids, and saponins (Xu et al. 2004), which are primarily derived from the plant kingdom, although other organisms also produce them (Thimmappa et al. 2014). They are synthesized from mevalonate via the 30-carbon intermediate, 2,3-oxidosqualene, which undergoes cyclization to form at least eleven major triterpene backbones (Thimmappa et al. 2014; Vincken et al. 2007). Subsequent decoration of the basic backbones of these precursor scaffold molecules involves a series of oxidation, glycosylation, and acylation modifications. Triterpenes are highly bioactive molecules with broad commercial applications in the pesticide, pharmaceutical, and cosmetic industries. They also contribute to biotic and abiotic stress tolerance in plants, playing a defensive role against diseases and pests, affecting plant growth and development, and regulating plant interactions with root-associated microorganisms (Moses et al. 2015; Liu et al. 2024).

Despite their high biotechnological potential, few studies have investigated the use of terpenes or terpenoids as BNIs, with contradictory results reported (Paavolainen et al. 1998; Bremner and McCarty 1988; Adamczyk et al. 2011). The most successful terpenoid identified as BNI is brachialactone, a tetracyclic diterpenoid isolated from root exudates of the pasture grass *Brachiaria* (*Brachiaria humidicola*) (Subbarao et al. 2009). Brachialactone acts both on AMO and hydroxylamine oxidoreductase, thereby affecting the second step of nitrification, which involves the transformation of hydroxylamine to nitrite, in pure cultures of *Nitrosomonas europaea*. Similarly, Ward et al. (1997) reported that monoterpenes inhibited *N. europaea* activity. To our knowledge, no studies have explored the BNI potential of triterpenoid chemistries.

Discovery of BNIs has mostly involved testing of inhibitory activity on cultures of a single genetically modified AOB strain, *N. europaea* (Otaka et al. 2022; Subbarao et al. 2006, 2013; Zakir et al. 2008). However, Leininger et al. (2006) reported the dominance of archaeal over bacterial ammonia oxidizers in several soils, while Aigle et al. (2019) showed that *Nitrosospira* sp. dominates the AOB community in agricultural soils. Consequently, Kaur-Bhambra et al. (2022) questioned the relevance of *Nitrosomonas europaea* as an indicator of BNI activity, highlighting the limitations of relying on a single strain bioassay, and proposed using a broader range of strains to better reflect the diversity of natural soil communities.

Recently, *in silico* tools have emerged as powerful approaches for predicting the inhibitory activity of chemical substances by analyzing structure–activity relationships or leveraging advanced techniques such as Graph Neural Networks (e.g. Ambe et al. 2024; Haga et al. 2023; Narayanaswamy et al. 2023; Shah et al. 2024). When trained on experimental data, models identify patterns and relationships, such as specific chemical structures or properties, that correlate with inhibitory effects (Sinha et al. 2023). Compared to traditional experimental approaches, predictive models offer significant advantages, including greater efficiency, scalability, and cost-effectiveness. This makes them invaluable for prioritizing candidate molecules, thereby accelerating the discovery and development of novel nitrification inhibitors.

In this context, this study aimed to evaluate the nitrification inhibition activity of 18 selected plant-derived triterpenoids, including tetra- and pentacyclic compounds, on a range of ecophysiologically and phylogenetically distinct soil-derived AOA and AOB strains in liquid cultures. We hypothesized that some of these triterpenoids may partially or fully inhibit the nitrification activity of the AOA and AOB strains used in this study, and this could point to structural leads associated with inhibition. To further pursue the latter, we applied an existing predictive model trained on AOB data to assess triterpenoid activity on AOA and AOB and subsequently refined it using an expanded dataset on AOA inhibition.

## Materials and methods

### Triterpenoids

High-purity analytical standards of 11-keto-beta boswellic acid (≥ 98%), 3-O-acetyl-11-keto-beta boswellic acid (≥ 99%), 3-O-acetyl-beta boswellic acid (≥ 98%), 3-O-acetyl-alpha boswellic acid (≥ 90%), beta boswellic acid (≥ 97%), asiatic acid (≥ 95%), madecassoside (≥ 97.5%), betulin (≥ 98%), betulinic acid (≥ 98%), chrysanthellin A (≥ 97%), chrysanthellin B (≥ 97%), cucurbitacin D (≥ 95%), cucurbitacin E (≥ 95%), cucurbitacin I (≥ 95%), echinocystic acid (≥ 98%), echinocystic acid-3-O-glucoside (≥ 98%), oleanolic acid (≥ 99%), and ursolic acid (≥ 98%) were purchased from EXTRASYNTHESE (France). More information on the tested compounds is provided in Table 1.

**Table 1.**
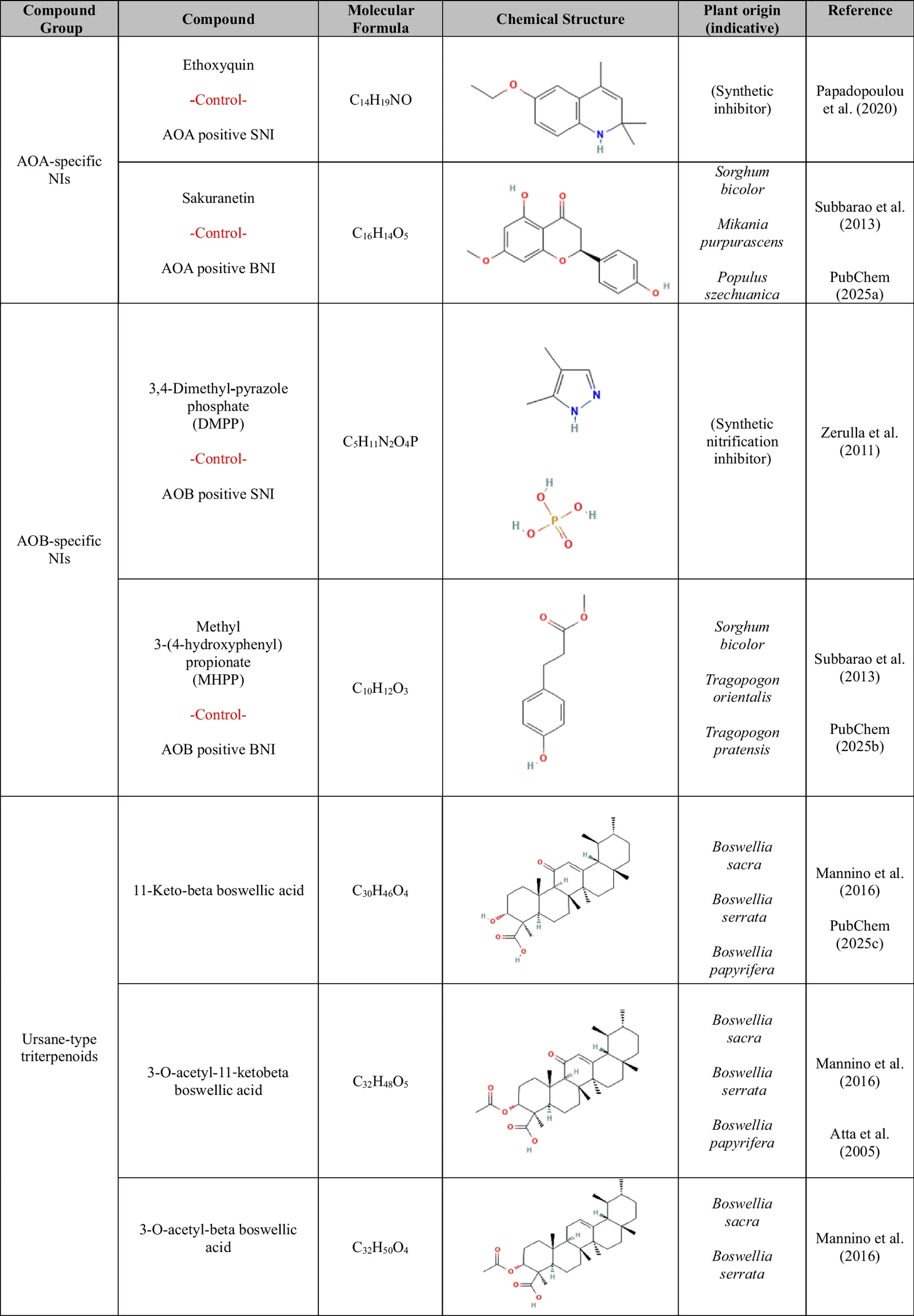

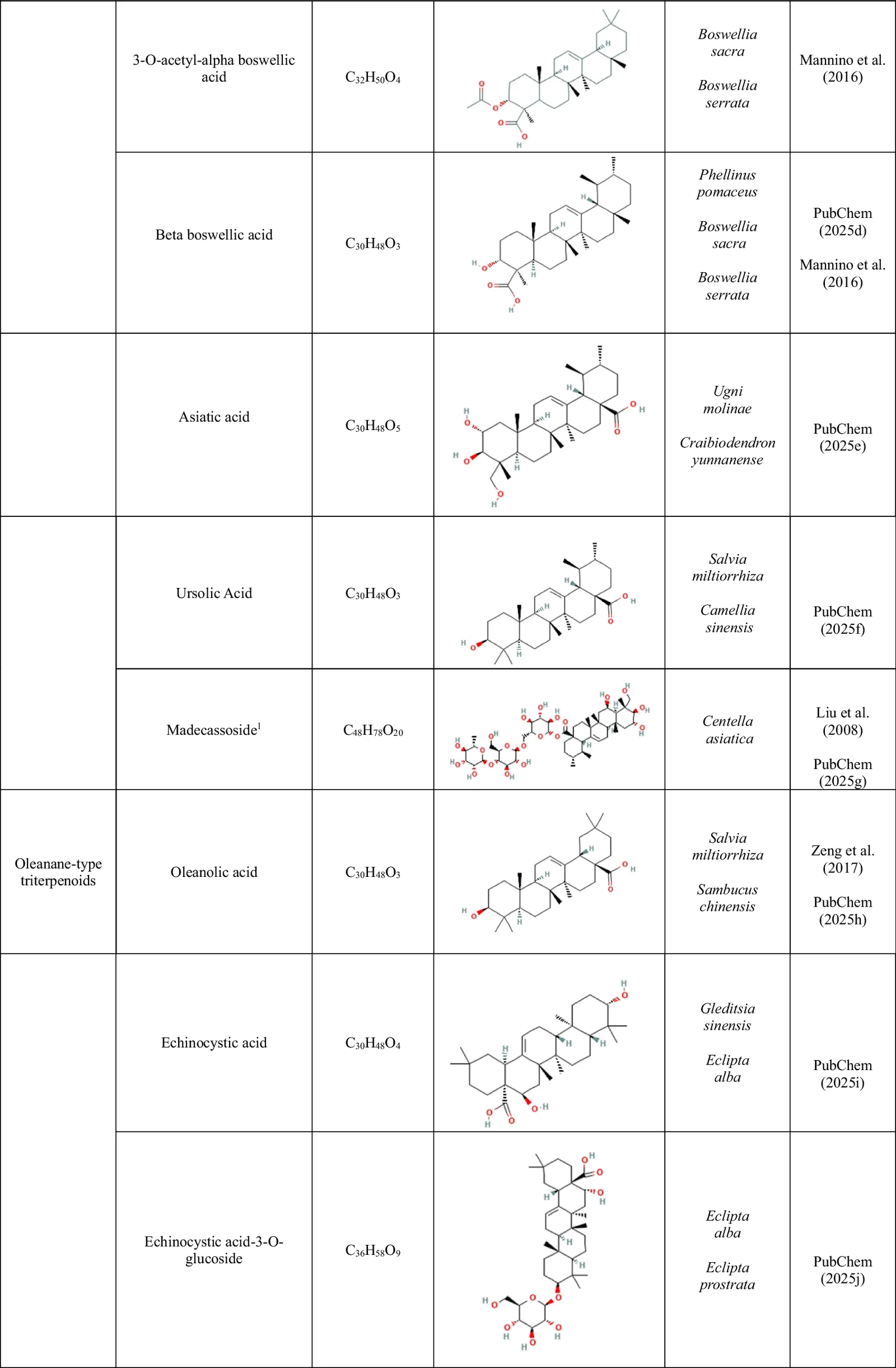

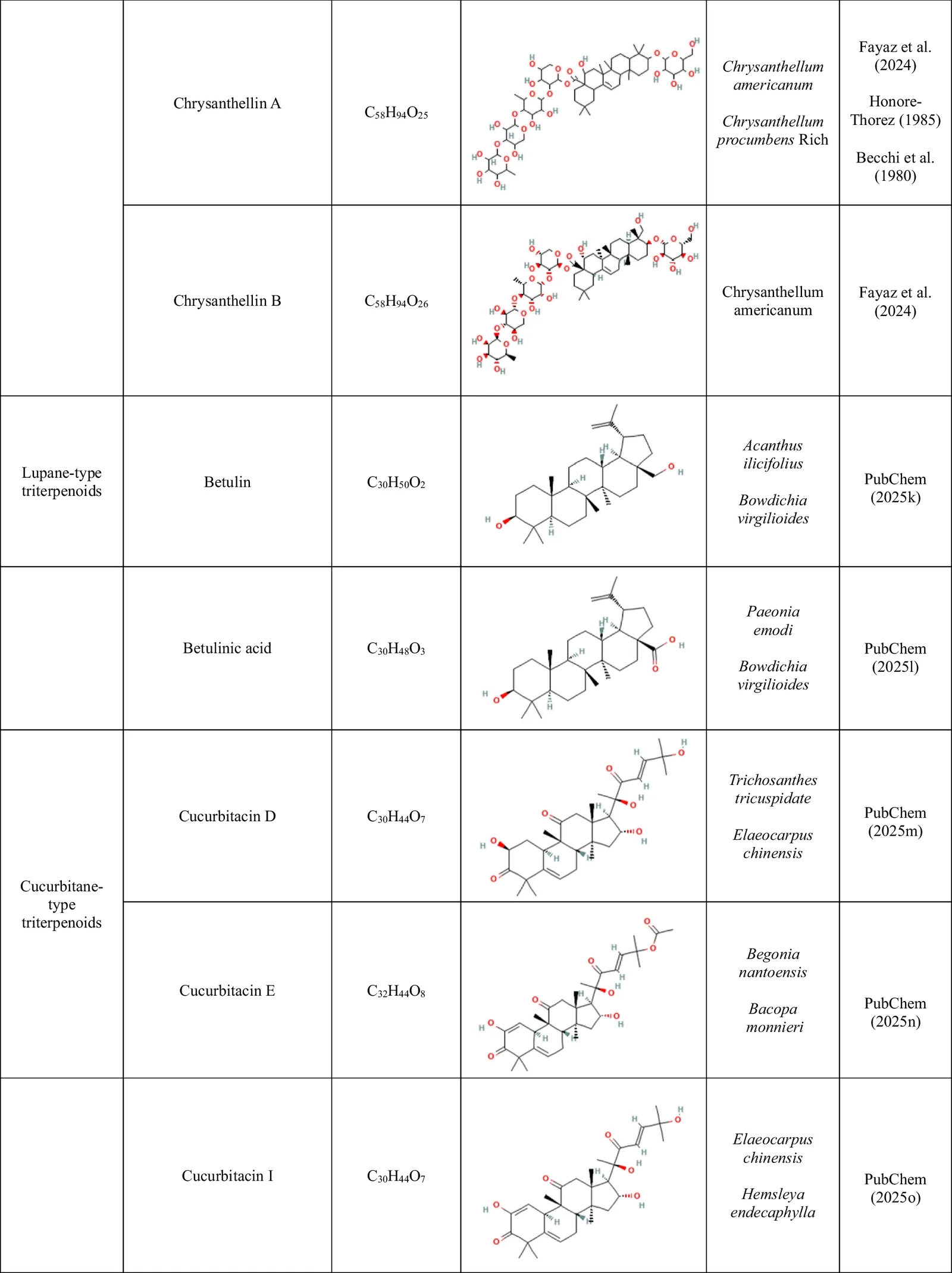
Molecular formulas, chemical structures, and key features of the compounds used in this study. The dataset includes triterpenoids, as well as known synthetic (SNIs) and biological nitrification inhibitors (BNIs) which served as positive controls for specific groups of ammonia-oxidizing microorganisms (ammonia-oxidizing bacteria, AOB; ammonia-oxidizing archaea, AOA). Ethoxyquin, included here as a novel potential SNI, was used as a positive control for AOA

^1^Madecassoside is a glycosylated ursane triterpenoid (saponin) derived from asiatic acid

### Microbial strains and growth conditions

Our inhibition assays included two AOB strains, *Nitrosomonas ureae* Nm10 and *Nitrosospira multiformis* ATCC 25196, and two AOA strains, *Nitrosotalea sinensis* ND2 (Lehtovirta-Morley et al. 2014) and *Nitrososphaera viennensis* EN76 (provided by Graeme Nicol, Université Claude Bernard Lyon, France, and Christa Schleper, University of Vienna, Austria). All strains were grown aerobically in the dark without shaking following previously established cultivation protocols for these strains (Tourna et al. 2011; Lehtovirta-Morley et al. 2014; Kolovou et al. 2023). Static incubation was applied to maintain conditions consistent with previous studies, using Duran bottles with sufficient headspace to ensure aerobic conditions without agitation. *N. multiformis* and *N. ureae* were grown at 28 °C, in Skinner and Walker’s medium (SW) (Skinner and Walker 1961) containing 1 mM NH_4_^+^ [(NH_4_)_2_SO_4_] and phenol red (0.5 mg L^−1^) as a pH indicator (pH 7.5–8.0). AOA *N. viennensis* and *N. sinensis* were incubated at 35 °C in a medium supplemented with 2 and 0.5 mM NH_4_^+^ (NH_4_Cl), respectively. The former was cultured in HEPES-buffered freshwater medium (pH 7.5 (Reyes et al. 2020), with slight modifications), while the latter was grown in MES-buffered freshwater (FW) medium (pH 5.2 (Lehtovirta-Morley et al. 2011)). For *N. viennensis*, the medium was modified by omitting the vitamin solution and adding 1 mM pyruvate to scavenge reactive oxygen species.

### Liquid culture inhibition assays

To determine the inhibitory activity of triterpenoids on the four ammonia-oxidizing microorganism (AOM) strains, controlled *in vitro* assays were performed in liquid batch cultures typically at two concentrations (20 and 5 mg L^−1^). For betulin, due to its very low water solubility, the concentrations tested were 5 and 2.5 mg L^−1^. Triterpenoids were added to the cultures as filter sterilized dimethyl sulfoxide (DMSO) solutions. Cultures were established in triplicate 100-mL Duran bottles for each concentration and triterpenoid combination. Each bottle contained 20 mL of growth medium inoculated with a 1% or 2% (vol/vol) transfer of exponentially growing cultures of the AOB and the AOA strains, respectively, following established cultivation protocols (Tourna et al. 2011; Bello et al. 2019; Papadopoulou et al. 2020; Bachtsevani et al. 2025). Control cultures of all strains were included with the addition of either DMSO (0.1% (vol/vol)) or sterile dH_2_O (negative control). The final concentration of DMSO in all cultures was 0.1% (vol/vol), which did not exert significant inhibitory effects to the isolates tested (Papadopoulou et al. 2020; Kaur-Bhambra et al. 2022).

Positive controls, i.e., those amended with SNIs and BNIs known to have an inhibitory effect on AOB and AOA, were also included. For this, analytical standards of ethoxyquin (96.9%, purchased from Sigma-Aldrich, Germany) and sakuranetin (> 95%, Syngenta Crop Protection AG, Switzerland) at two concentrations (20 and 5 mg L^−1^) were used as positive inhibitory controls of the AOA strains, based on previous studies by Papadopoulou et al. (2020) and Kolovou et al. (2023). Similarly, analytical standards of 3,4-dimethylpyrazole phosphate (DMPP) (≥ 98%, purchased from Cayman Chemical, USA) and methyl 3-(4-hydroxyphenyl)propionate (MHPP) (99.2%, purchased from BLDpharm, China) at two concentrations (20 and 5 mg L^−1^) were used as positive inhibitory controls for the AOB strains (Papadopoulou et al. 2020; Kaur-Bhambra et al. 2022; Kolovou et al. 2023). For MHPP, an additional 100 mg L^−1^ concentration was tested to achieve (according to preliminary data) full inhibition of both *N. multiformis* and *N. ureae* (Kaur-Bhambra et al. 2022; Kolovou et al. 2023).

All triterpenoids and control compounds (negative or positive) were added to batch cultures at the start of the exponential growth phase. Upon inoculation, cultures were sampled at regular intervals (once or twice daily) to assess the effect of triterpenoids on the tested AOM strains by measuring changes in nitrite concentrations. Nitrite levels were determined colorimetrically at 540 nm using a 96-well plate assay with diazotization and coupling with Griess reagent (Shinn 1941).

### *In silico* prediction of triterpenoids as potential biological nitrification inhibitors

We further explored whether the inhibitory potential of triterpenoids could be predicted *in silico*. For this, we applied an existing Graph Neural Network (GNN) model originally trained on AOB inhibition data (Zhang et al. 2025). The model was first applied to assess whether triterpenoids were predicted to inhibit the AOA and AOB strains studied here, and it was then refined using an expanded dataset on *N. sinensis* inhibition, the only strain for which a comprehensive dataset was available to support model training, based on graph representations of molecules. The dataset used to train the model consisted of data produced upon tests of *N. sinensis* with the 18 triterpenoids, 6 synthetic nitrification inhibitors (Papadopoulou et al. 2020), 11 biological nitrification inhibitors with diverse chemical structures (Kolovou et al. 2023), 24 pesticides from different chemical categories (Bachtsevani et al. 2025; Bachtsevani et al. unpublished data), and 5 anthelmintic compounds (unpublished data). Ιn this refined model, compounds with EC_50_ values below 20 mg L^−1^ were considered positive, while those with higher values were considered negative.

To this end, a GNN was built to classify compounds as positive or negative using their Simplified Molecular Input Line Entry System (SMILES) representations as input. The dataset was split into 80% for training, 10% for validation, and 10% for testing. To ensure an unbiased evaluation of model performance, we conducted five independent random splits of the data. Additionally, to augment the dataset and provide the model with more diverse learning examples, we applied several transformations to the SMILES to generate their tautomers with RDKit (RDKit 2025). These transformations included keto-enol, imine-enamine, amide-imidic acid, lactam-lactim, pyridine-pyridol, heteroatom proton transfer, protonation/deprotonation of nitro groups, thione-thiol, and azole tautomerism. After these transformations, the dataset comprised 335 SMILES. All tautomeric forms of a compound were assumed to share the same inhibition potential. The model performance was re-evaluated with the augmented dataset. Data leakage was carefully avoided by ensuring that tautomers of the same compound were assigned exclusively to one of the training, validation, or test sets during data splitting.

The GNN architecture was adapted from Zhang et al. (2025) and fine-tuned from a pre-trained version on a mechanistically relevant log P dataset containing 14,050 compounds (Ulrich et al. 2021). Message passing was implemented through sequential Graph Attention Network Convolution (GATConv) layers in PyTorch (Veličković et al. 2018). A multi-head attention layer was added to enhance the ability of the model to capture relationships among different atoms. The final molecular embeddings were obtained by aggregating node features with global max pooling and global average pooling, followed by two fully connected layers for molecular property classification. Attention weights from the multi-head attention layer were extracted and corrected following Zhang et al. (2025), and Shapley values were used to quantify the contribution of substructures potentially responsible for nitrification inhibition.

### Data and statistical analysis

Ammonia oxidation inhibition (AOI) was expressed as the percentage inhibition of AOM activity relative to the DMSO control treatment after triterpenoid addition. The value recorded immediately before triterpenoid addition was subtracted from subsequent measurements to account for pre-treatment activity. Data processing and visualisation were conducted with the RStudio software (R Core Team 2020). In RStudio, plot generation utilized the “ggplot2” package v3.5.0 (Wickham 2016) unless otherwise declared. In the duration of the analyses, the R packages “readxl” v1.4.3 (Wickham and Bryan 2023), “dplyr” v1.1.4 (Wickham et al. 2023), and “tibble” v3.2.1 (Müller and Wickham 2023) have been used for general data processing purposes, and the “ggplot2” v3.5.0 (Wickham 2016) and “ggpubr” v0.6.0 (Kassambara 2023a) R packages have been used for the generation of figures. For data distribution validation, the Shapiro–Wilk normality test with the supplementary histogram and q-q plot was conducted using the “stats” base package in the R Software. For comparing nitrite concentration for all treatments against the control for all four strains, *t*-tests were performed using the “rstatix” v0.7.2 (Kassambara 2023b) package. AOI% of the triterpenoids against the four different AOM reporter strains was explored separately for the two applied concentrations (5 mg L^−1^ and 20 mg L^−1^ or 5 and 2.5 mg L^−1^ for betulin), with comparisons employing a Kruskal–Wallis test followed by Dunn’s post hoc test with a Bonferroni *p*-value adjustment method, utilizing the “agricolae” v1.3.7 (De Mendiburu 2023) and the “rstatix” v0.7.2 (Kassambara 2023b) packages. The effect of the triterpenoids on the two AOM domains was investigated again separately for the two applied concentrations with a Wilcoxon test using the integrated function of the “ggplot2” v3.5.0 R package (Wickham 2016). The heatmap of triterpenoid AOI% values was generated with the “ComplexHeatmap” v2.14.0 (Gu 2016) and “dendextend” v1.17.1 (Galili 2015) R packages, with both column and row dendrograms based on hierarchical clustering and Euclidean distances.

## Results

### The inhibition of known BNIs and SNIs on the activity of AOA and AOB strains

**Fig. 1:**
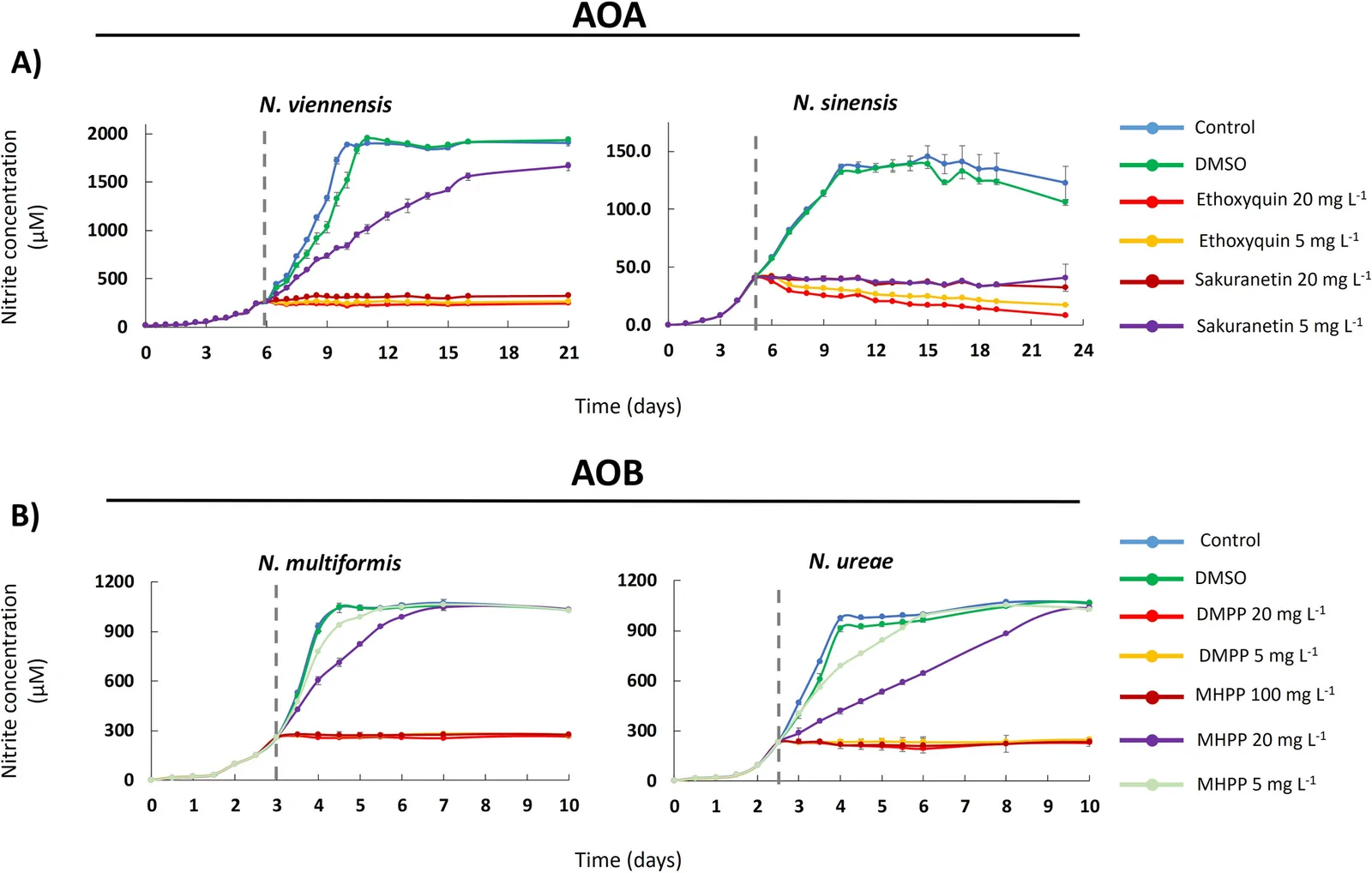
The inhibition of selected synthetic (SNIs) and biological nitrification inhibitors (BNIs) on the activity of ammonia-oxidizing archaea (AOA) and bacteria (AOB), assessed by nitrite production. **A** Ethoxyquin and sakuranetin on *N. viennensis* and *N. sinensis*. **B** 3,4-dimethylpyrazole phosphate (DMPP) and methyl 3-(4-hydroxyphenyl)propionate (MHPP) on *N. multiformis* and *N. ureae*. All compounds were tested at 5 and 20 mg L^−1^, with MHPP also tested at 100 mg L^−1^. Error bars represent the standard error of the mean of triplicate cultures. The vertical dashed line indicates the time at which the compounds were added to the cultures

The effects of the known SNIs and BNIs, tested as positive controls, on the nitrification activity of AOA and AOB strains are shown in Fig. 1. Ethoxyquin, at both concentrations, induced complete inhibition (AOI ≥ 100%, *p* < 0.001) of both AOA strains (Fig. 1 and Supplemental Table S1). Sakuranetin showed complete inhibition (AOI ≥ 96.3%, *p* < 0.001) of both AOA strains at the highest concentration, while at the lower concentration, it fully inhibited *N. sinensis* (AOI ≥ 98.5%, *p* < 0.05), but not *N. viennensis* which showed recovery at the end of the incubation (Fig. 1 and Supplemental Table S1). DMPP induced a complete (AOI ≥ 97.6%, *p* < 0.001) and persistent inhibition on both AOB strains at both concentrations tested (Fig. 1 and Supplemental Table S1). The effect of MHPP varied depending on the strain and the concentration tested. At 100 mg L^−1^, it induced complete inhibition of both AOB strains (AOI ≥ 97.6%, *p* < 0.001) (Fig. 1 and Supplemental Table S1), whereas at 20 and 5 mg L^−1^, it caused only transient inhibition (*p* < 0.001) of *N. multiformis* (AOI ≥ 46.3% and ≤ 19.3%) and *N. ureae* (AOI ≥ 72.3% and ≤ 32.9%), respectively (Fig. 1 and Supplemental Table S1).

### The inhibition of triterpenoids on the activity of AOA and AOB strains

**Fig. 2:**
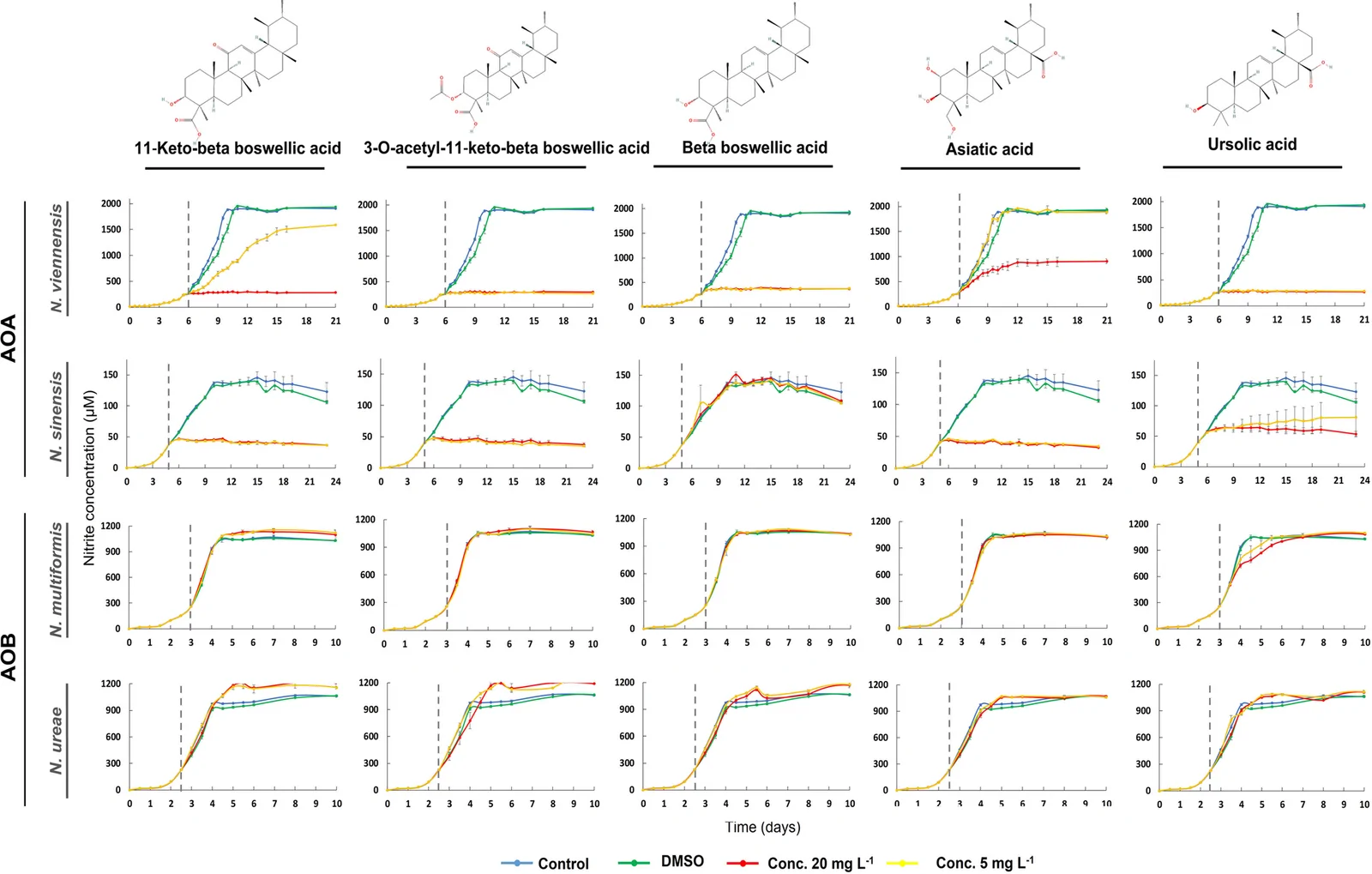
Effect of the subset of ursane-type triterpenoids showing inhibitory activity (11‐keto-beta boswellic acid, 3-O-acetyl-11‐keto-beta boswellic acid, beta boswellic acid, asiatic acid and ursolic acid), on ammonia-oxidizing archaea (*Nitrososphaera viennensis* and *Nitrosotalea sinensis*) and ammonia-oxidizing bacteria (*Nitrosospira multiformis* and *Nitrosomonas ureae*). Compounds were tested at 5 and 20 mg L^−1^. Activity was assessed by monitoring nitrite production. Error bars represent the standard error of the mean of triplicate cultures. The vertical dashed line indicates the time at which the compounds were added to the cultures

The triterpenoids tested in this study include 15 pentacyclic esters, belonging to the main ursane, oleanane, and lupane types, as well as three tetracyclic cucurbitane-type triterpenoids (Table 1). The effects of ursane-type triterpenoid derivatives are presented in Fig. 2 (subset showing inhibitory activity) and Supplementary Fig. S1 (subset showing no inhibitory activity). 11-keto-beta boswellic acid caused complete inhibition (AOI ≥ 94.0%, *p* < 0.001) of both AOA strains at the high concentration and of *N. sinensis* at the low concentration (AOI ≥ 95.4%, *p* < 0.001). For *N. viennensis*, inhibition at the low concentration was significant but transient (AOI = 61.5%, *p* < 0.001) (Supplemental Table S1). 3-O-acetyl-11‐keto-beta boswellic acid induced complete (AOI ≥ 94.2%, *p* < 0.001) and persistent inhibition of both AOA strains at both concentrations tested (Supplemental Table S1). Beta-boswellic acid completely inhibited *N. viennensis* (AOI ≥ 93.3%, *p* < 0.001) but had not significant effect on *N. sinensis* (Fig. 2, Supplemental Table S1). Asiatic acid completely inhibited *N. sinensis* at both concentrations tested (AOI ≥ 97.9%, *p* < 0.001) (Fig. 2, Supplemental Table S1) and exerted a concentration-dependent effect on *N. viennensis* (AOI ≥ 61.7%, *p* < 0.001 at the high concentration only). Ursolic acid induced complete inhibition (AOI ≥ 99.1%, *p* < 0.001) of *N. viennensis* at both tested concentrations (Supplemental Table S1) and significantly reduced the activity of *N. sinensis* (70.3 and 74.3% for the low and high concentration respectively, *p* < 0.05). By contrast, other ursane-type derivatives, such as 3-O-acetyl-beta boswellic acid, 3-O-acetyl-alpha boswellic acid, and madecassoside did not significantly inhibit either AOA strain (*p* ≥ 0.05) (Supplementary Fig. S1 and Supplemental Table S1). None of the boswellic acid analogues or asiatic acid significantly affected AOB strains (*p* ≥ 0.05) (Fig. 2, Supplementary Fig. 1, Supplemental Table S1).

**Fig. 3:**
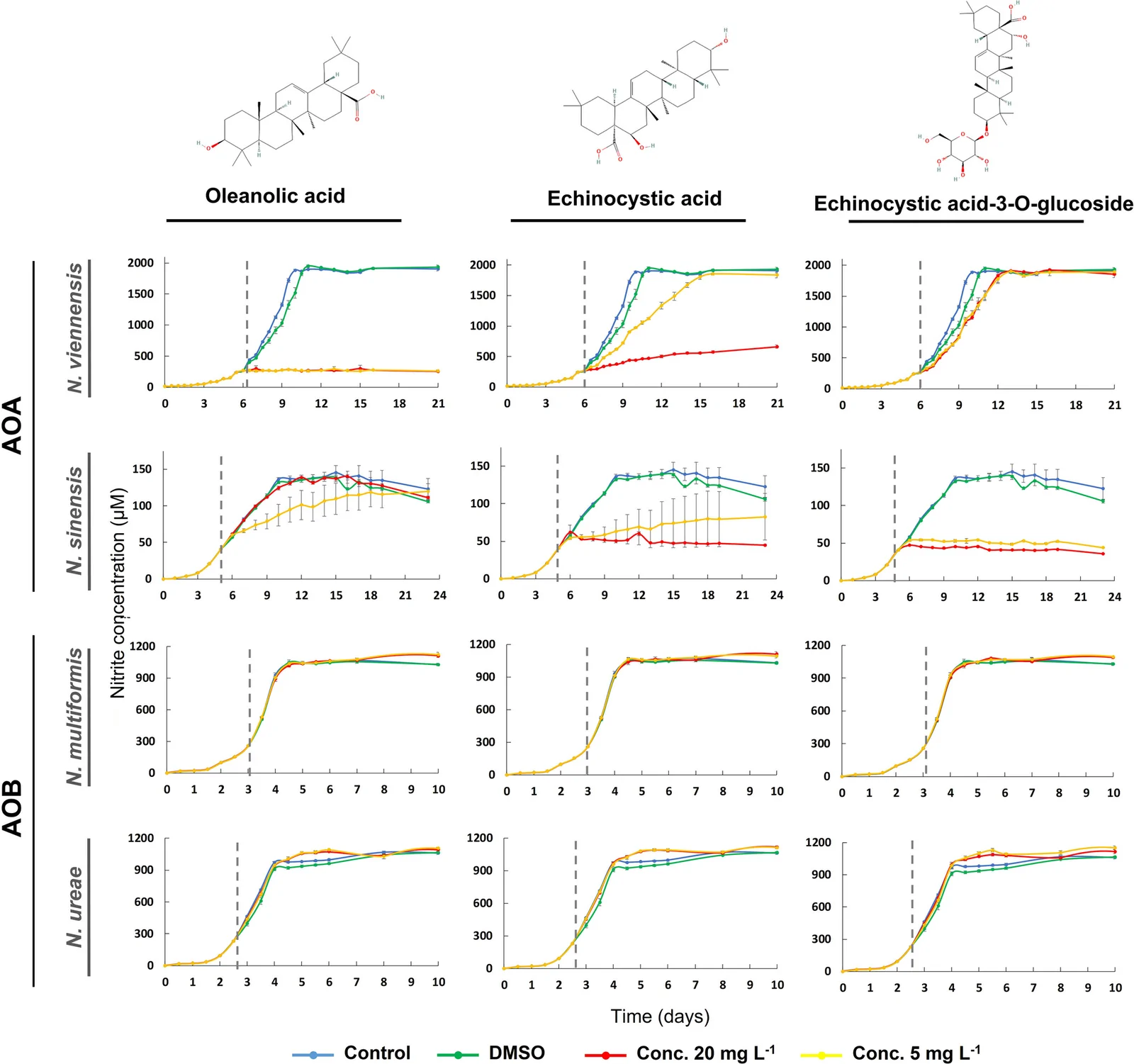
Effect of the subset of oleanane-type triterpenoids showing inhibitory activity (oleanolic acid, echinocystic acid, and echinocystic acid-3-O-glucoside) on ammonia-oxidizing archaea (*Nitrososphaera viennensis* and *Nitrosotalea sinensis*) and ammonia-oxidizing bacteria (*Nitrosospira multiformis* and *Nitrosomonas ureae*). Compounds were tested at 5 and 20 mg L^−1^. Activity was assessed by monitoring nitrite production. Error bars represent the standard error of the mean of triplicate cultures. The vertical dashed line indicates the time at which the compounds were added to the cultures

The effects of oleanane-type triterpenoid derivatives are shown in Fig. 3 (subset showing inhibitory activity) and Supplementary Fig. S2 (subset showing no inhibitory activity). Oleanolic acid induced complete inhibition (AOI = 100%, *p* < 0.001) of *N. viennensis* at both tested concentrations, while its effect on *N. sinensis* was marginal and transient (Fig. 3 and Supplemental Table S1). Echinocystic acid induced strong and persistent inhibition of both AOA strains at the high concentration (AOI ≥ 76.3%, *p* < 0.001) (Supplemental Table S1), whereas its low concentration caused significant but transient inhibition of *N. viennensis* (AOI = 49.3%, *p* < 0.001) and *N. sinensis* (AOI = 74.9%, *p* < 0.05) (Supplemental Table S1). Its glucosylated derivative, echinocystic acid-3-O-glucoside, strongly inhibited *N. sinensis* at both tested concentrations (AOI ≥ 86.0%, *p* < 0.001), but exerted only transient and weak inhibition on *N. viennensis* (*p* < 0.05, AOI = 28.8 and 30.5%, respectively, at higher and lower concentration) (Supplemental Table S1). None of these compounds significantly inhibited the AOB strains (Fig. 3, Supplemental Table S1). Other oleanane-type derivatives, such as chrysanthellin A and chrysanthellin B, had no consistent inhibitory effect on either AOA or AOB strains (Supplementary Fig. S2 and Supplemental Table S1).

The effects of lupane-type triterpenoid derivatives (betulin and betulinic acid) on AOA and AOB strains are shown in Supplementary Fig. S3. Betulin induced a significant but transient inhibition of *N. sinensis* at both concentrations tested (5 and 2.5 mg L^−1^) (AOI ≥ 30.5%, *p* < 0.001) (Supplemental Table S1). In contrast, only marginal inhibition was observed for *N. multiformis* at both tested concentrations (AOI ≤ 8.3%, *p* < 0.05), while no significant inhibitory effect (*p* ≥ 0.05) was detected for *N. ureae* and *N. viennensis*. Betulinic acid exerted a weak and transient inhibitory effect on *N. sinensis* (AOI = 13.1%, *p* < 0.01) but did not inhibit any of the other tested AOM strains (Supplementary Fig. S3 and Supplemental Table S1).

The effects of cucurbitacins D, E, and I on AOA and AOB strains are shown in Supplementary Fig. S4. Cucurbitacin D and I caused only low and transient inhibition of *N. sinensis* (*p* < 0.05, 6.3% and 11.1%, respectively). At the high concentration, cucurbitacin I also transiently inhibited *N. viennensis* (AOI = 17.1%, *p* < 0.01) (Supplemental Table S1). Cucurbitacin E induced significant but transient inhibition of *N. viennensis* (AOI = 66.8%, *p* < 0.001) and *N. sinensis* (AOI = 14.6%, *p* < 0.01) at the high concentration. At lower concentration, it imposed weaker and transient inhibition of *N. sinensis* (AOI = 6.6%, *p* < 0.05). None of the cucurbitacins tested showed significant inhibitory effects (*p* > 0.05) on the AOB strains (Supplementary Fig. S4 and Supplemental Table S1).

### Inhibitory patterns of triterpenoids on AOA and AOB strains

When collectively analyzed, triterpenoids showed significantly higher inhibitory effects on AOA compared to AOB strains (Wilcoxon test *F*-statistic = 9402, *p* < 0.001 and 8859, *p* < 0.001 for the high and the low dose, respectively) (Supplementary Fig. S5). At the strain level, the Kruskal–Wallis analysis verified the significantly higher (*χ*^2^ = 64.71, *p* < 0.001) inhibitory activity of triterpenoids on AOA strains compared to AOB strains; however, no significant differences between the two AOA and the two AOB strains tested were observed (Supplementary Fig. S6).

**Fig. 4:**
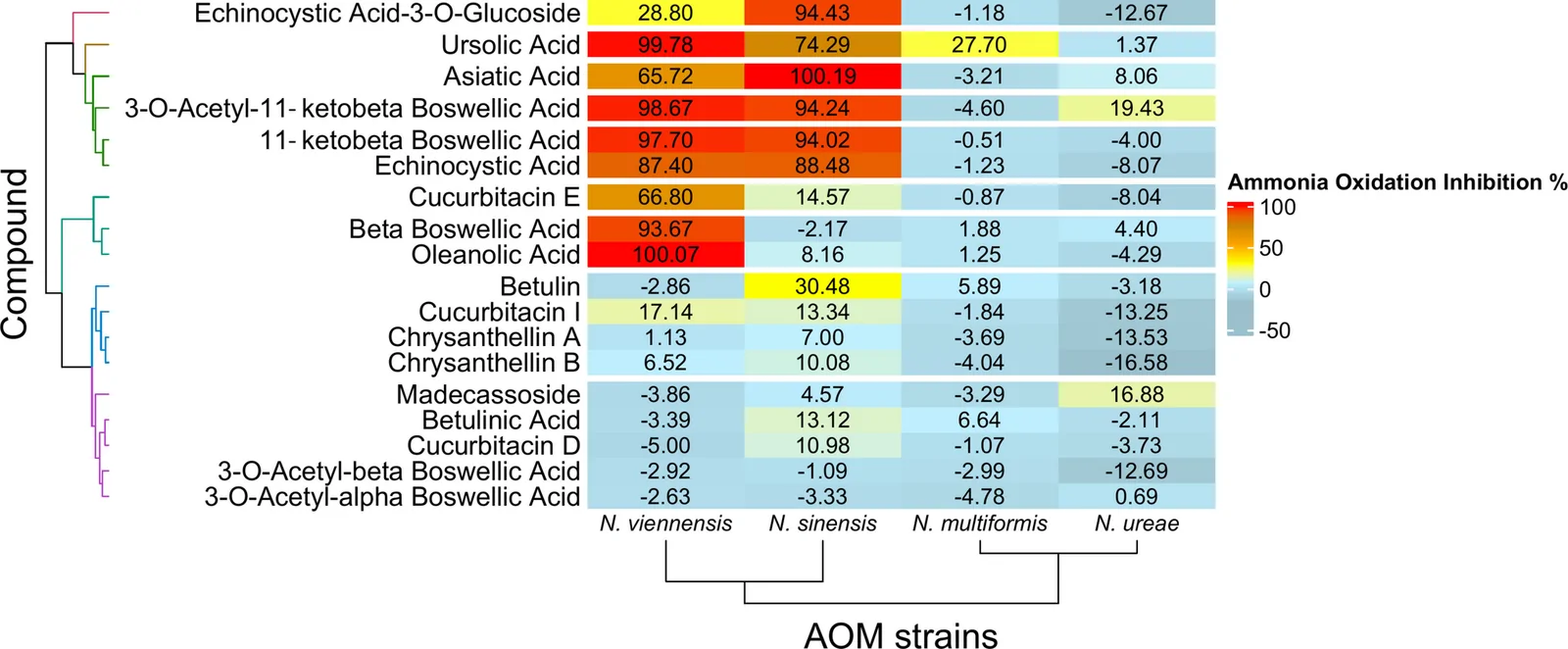
Comparison of triterpenoid inhibitory activity across the tested ammonia-oxidizing microbial (AOM) strains, including ammonia-oxidizing archaea (*Nitrososphaera viennensis* and *Nitrosotalea sinensis*) and ammonia-oxidizing bacteria (*Nitrosospira multiformis* and *Nitrosomonas ureae*). The heatmap illustrates the mean ammonia oxidation inhibition (AOI%) values for each triterpenoid across the four tested strains. Box colours represent AOI% values, as indicated by the scale bar. Dendrograms for both rows and columns were generated by hierarchical clustering of AOM strains and triterpenoids based on Euclidean distances

Hierarchical clustering, based on the inhibition percentages imposed by the high concentration of the tested compounds, was used to group strains and triterpenoids (Fig. 4). AOA and AOB strains formed two distinct clusters, reflecting their largely different sensitivities to the triterpenoids tested. Regarding triterpenoids, based on their inhibitory patterns, they were grouped into four major clusters (Fig. 4). The first cluster consists of echinocystic acid, its glucosilated derivative echinocystic acid-3-O-glucoside, asiatic acid, 11-ketobeta boswellic acid, 3-O-acetyl-11‐ketobeta boswellic acid, and ursolic acid, all exhibiting strong and consistent inhibition on both AOA strains (close to or above 80%), with the last two compounds also showing weak inhibition activity towards AOB strains. Exceptions include echinocystic acid-3-O-glucoside and asiatic acid, which showed 28.8% and 65.7% inhibition of *N. viennensis*, respectively. The second cluster comprises oleanolic acid, cucurbitacin E, and beta boswellic acid, which exhibited high inhibition activity (> 65%) primarily against *N. viennensis*. The third cluster includes chrysanthellin A and B, betulin, and cucurbitacin I, which exhibited moderate inhibition (≤ 30%) of AOA strains, and had no effect on AOB strains. The fourth cluster included madecassoside, betulinic acid, cucurbitacin D, 3-O-acetyl-beta boswellic acid, and 3-O-acetyl-alpha boswellic acid, which showed little or no inhibition of any of the AOM strains tested.

### *In silico* prediction of triterpenoids as potential biological nitrification inhibitors

**Fig. 5:**
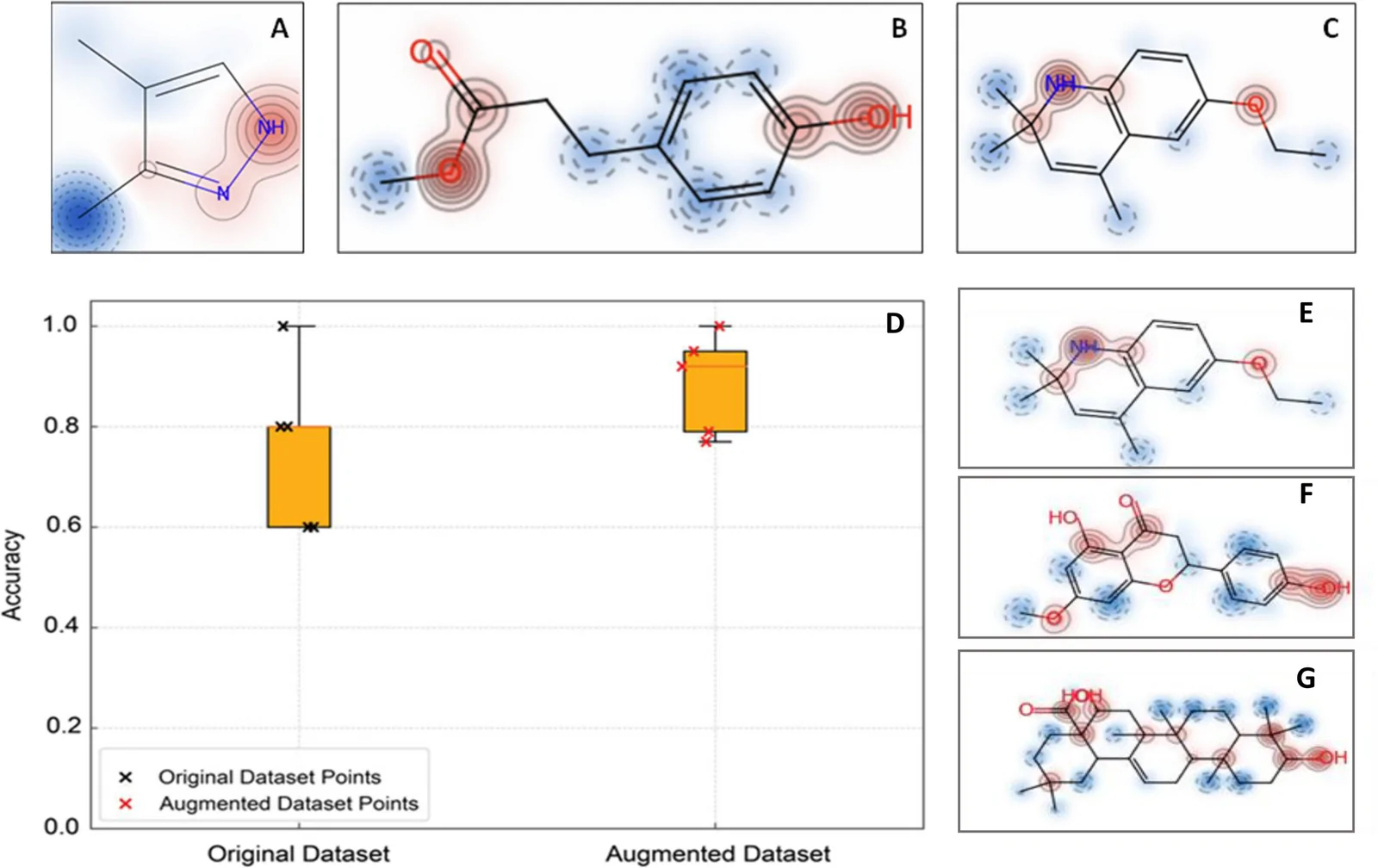
Model predictions of inhibitory activity and structural feature attribution. **A**–**C** Examples of molecules predicted as positive by the AOB-trained model: **A** 3,4-dimethylpyrazole phosphate (DMPP), **B** methyl 3-(4-hydroxyphenyl)propionate (MHPP), and **C** ethoxyquin. **D** Prediction accuracy of the AOA-trained model based on the original dataset (64 SMILES) and the extended dataset (335 SMILES); bars represent the average prediction accuracy for each dataset. **E**–**G** Examples of molecules predicted as positive by the AOA-trained model, including ethoxyquin (**E**), sakuranetin (**F**), and an oleanolic acid tautomer (**G**). Red regions indicate substructures receiving high attention, whereas blue regions indicate substructures receiving low attention. Substructures with higher attention weights are considered more important for the prediction of nitrification inhibition (NI) activity

Predictions for the tested triterpenoids were first generated using the AOB-trained model. Supplemental Table S2 shows the predictions from this model which correctly identified the known NIs DMPP, MHPP, and ethoxyquin as positive for AOB and AOA inhibition respectively, while sakuranetin was misclassified as negative for AOA inhibition. All triterpenoids were predicted as negative for both AOB and AOA strains. For the compounds predicted as positive, the model highlighted structural alerts potentially associated with their nitrification inhibitory activity including an amine group in DMPP (Fig. 5A), a methyl ester and a hydroxyl group in MHPP (Fig. 5B), and an amine plus an ethoxy- group in ethoxyquin (Fig. 5C).

A second model with the same architecture as the AOB one was then trained on an *N. sinensis* dataset. Using the dataset of 64 compounds, the average prediction accuracy reached 0.76, which increased to 0.89 when the model was trained with the augmented dataset comprising 335 SMILES (Fig. 5D). These accuracy values reflect overall model performance based on repeated train/test splits and should not be interpreted as independent predictions for the triterpenoids, which were part of the training data. Instead, the improved performance indicates that the inclusion of AOA data enables the model to generalize better across the broader compound set. Using attention mechanisms and Shapley values, the model identified several substructures as potential contributors to the nitrification inhibitory activity, including O = C(-OH)-C–C-OH, C-NH-c, c–O–C, c–OH, and O-c–c-C = O, where “C” denotes aliphatic carbon and “c” stands for aromatic carbon (Fig. 5E–G).

## Discussion

Triterpenoids are secondary plant metabolites that exhibit a wide range of biological activities; however, their potential as BNIs has not been largely explored. Here, we investigated the effects of a range of structurally diverse triterpenoids on two AOA and two AOB strains, chosen for their ecological importance and representativeness in soil ecosystems (Alves et al. 2018; Zorz et al. 2018). The consistent and selective inhibitory activity of known nitrification inhibitors towards the tested AOB (DMPP and MHPP) and AOA (ethoxyquin and sakuranetin) strains, in line with previous studies (Papadopoulou et al. 2020; Kaur – Bhambra et al. 2022; Kolovou et al. 2023), reinforced the reliability of our assay for identifying molecules with NI activity.

Based on our assays, certain triterpenoids, notably the ursane-type derivatives ursolic acid, 11-keto-beta-boswellic acid, 3-O-acetyl-11‐ketobeta boswellic acid, as well as the oleanane-type derivative echinocystic acid, were identified as efficient inhibitors of the AOA strains. These triterpenoids exhibited inhibitory activity on AOA that was similar to or higher than that of sakuranetin, a BNI used in our study as a model compound. Interestingly, activity was largely confined to ursane-type triterpenoids, while neither of the two lupane-type compounds inhibited any of the four AOM strains. Similarly, relatively small structural modifications appeared sufficient to either enhance (i.e., the C11-carbonylation of boswellic acid) or abolish (i.e., the glysosylation of echinocystic acid) the inhibitory effect of triterpenoid molecules. Similar observations linking specific chemical moieties to differences in NI efficiency have been reported previously (Egenolf et al. 2020). Based on these data and datasets available from previous studies, we employed GNN analyses to identify chemical features that could be associated with inhibitory activity on AOM. Our analysis indicated hydroxyl- and carboxyl-containing motifs, as well as glucoside linkages, as potential contributors to triterpenoid inhibitory activity, in line with earlier reports (Ramachandran et al. 2014; Kim et al. 2019) and with our finding that glucosylation of echinocystic acid reduced its inhibitory effect compared to the aglycone. Yet the initial AOB-trained model (Zhang et al. 2025) could not reproduce the strong inhibitory effects of triterpenoids on AOA, despite correctly classifying known inhibitors such as DMPP, MHPP, and ethoxyquin and capturing the lack of triterpenoid activity on AOB. Retraining the model with an *N. sinensis* dataset substantially improved predictive performance, underscoring the importance of dataset size and diversity (e.g., AOB vs. AOA) for enhancing model generalization across diverse compound classes. Expanding the dataset with additional AOA *in vitro* results will be critical to strengthen predictive reliability and enable earlier discovery of bioactive BNIs.

Similar graph-based deep learning approaches have shown increasing promise in chemical bioactivity modelling (e.g., Stokes et al. 2020; Jiang et al. 2021). However, as in our case, their reliability depends strongly on the size, diversity, and representativeness of the training datasets. Τhe relatively small number of experimentally validated nitrification inhibitors and the predominance of AOB-derived data may have limited prediction accuracy of our model for the phylogenetically distinct AOA. This is consistent with previous reports showing reduced transferability of machine learning models between biological systems with divergent enzymatic pathways (Cui et al. 2025). Nevertheless, the successful identification of structural motifs consistent with experimental evidence suggests that the model captures biologically meaningful patterns rather than random correlations. Taken together, these findings support the potential of *in silico* screening as a complementary tool to guide the discovery of novel BNIs, provided that future models are trained on more extensive and taxonomically balanced datasets.

Although previous studies on terpenes reported mixed or partial inhibition of nitrification (Bremner and McCarty 1988; Paavolainen et al. 1998; Adamczyk et al. 2011), they did not investigate potential differences in activity against AOB and AOA—a feature that our study highlights. One of the most distinct outcomes of our study was the selective inhibitory activity of certain triterpenoids on AOA compared to AOB. Several recent culture-based studies suggested that the majority of plant-derived inhibitors like 1,9-decanediol, sakuranetin, linoleic acid, linolenic acid, methyl linoleate (Kaur–Bhambra et al. 2022), 2-methoxy-1,4-naphthoquinone, and caffeic acid (Kolovou et al. 2023) exert stronger inhibition on AOA than on AOB. The higher sensitivity of AOA to triterpenoids compared to AOB may result from fundamental biochemical and physiological differences between these two microbial groups (Koops and Pommerening-Röser 2005; Schleper and Nicol 2010), including variations in membrane composition and organization (Valentine 2007). In contrast to bacterial membranes composed of ester-linked fatty acids, archaeal membranes are built from ether-linked, methyl-branched isoprenoid chains attached to glycerol-1-phosphate backbones (Koga and Morii 2005). In particular, the AOA strains used in this study possess membranes characterized by a complex lipid inventory of glycerol dibiphytanyl glycerol tetraethers (GDGTs), glycerol diphytanyl diethers (archaeols), and related isoprenoid lipids. Among these, GDGTs, particularly crenarchaeol, which is specific to *Nitrososphaerota*, represent a defining structural component of AOA membranes (Pitcher et al. 2010; Stieglmeier et al. 2014; Lehtovirta-Morley et al. 2024). These tetraether lipids can form densely packed membrane structures with physicochemical properties that differ substantially from bacterial bilayers (Siliakus et al. 2017). Such differences in lipid chemistry and membrane architecture may influence how bulky hydrophobic molecules, such as triterpenoids, partition into and distribute within archaeal membranes, potentially modifying membrane integrity or altering the local microenvironment of membrane-associated enzymes such as AMO.

In addition to membrane-level effects, the strong inhibitory activity of several triterpenoids (e.g., 11-keto-beta-boswellic acid, 3-O-acetyl-11-keto-beta-boswellic acid, echinocystic acid) on AOA is consistent with a possible mechanism involving inhibition of 3-hydroxy-3-methylglutaryl-CoA reductase (HMGCR), a key enzyme in the archaeal mevalonate pathway (Lam and Doolittle 1992; Miller and Wolin 2001), although no direct biochemical evidence was generated in the present study. Notably, eukaryotes and most archaea share the same class of HMGCR (Cerqueira et al. 2016), and their mevalonate pathways are also comparable (Vinokur et al. 2014), unlike bacteria that utilize an alternative pathway for cell membrane biosynthesis (Jain et al. 2014). Simvastatin, a well-known HMGCR inhibitor, was recently reported as a selective inhibitor of a range of AOA strains, including *N. sinensis* and *N. viennensis* (Zhao et al. 2020). Interestingly, acidophilic strains such as *N. sinensis* and *Nitrosotalea devanaterra* were inhibited at 3.3 mg L^−1^, whereas neutrophilic strains *N. viennensis* and *Candidatus* Nitrosocosmicus franklandianus required 40 mg L^−1^, suggesting that pH-dependent physiological traits may influence sensitivity, possibly through differences in membrane lipid composition and enzyme stability between acidophilic and neutrophilic AOA (Lehtovirta-Morley et al. 2016; Kerou et al. 2016). Still, the potential differential behaviour of these molecules under different pH conditions (i.e., changes in ionization state and polarity) may also modulate their interaction with archaeal membranes or intracellular targets and therefore cannot be excluded.

Several triterpenoids tested here have previously shown potential or confirmed inhibitory activity on HMGCR, including ursolic acid (Kim et al. 2019), asiatic acid (Ramachandran et al. 2014), oleanolic acid (Luo et al. 2024), cucurbitacin E (Sangande et al. 2023), and boswellic acids (Sanchez et al. 2022). This inhibitory mechanism may only partially explain the effects of some triterpenoids on the two AOA strains. Deviations from this pattern suggest that additional strain-specific mechanisms are likely involved. For example, oleanolic acid did not inhibit *N. sinensis* at 20 mg L^−1^, but persistently inhibited *N. viennensis*, suggesting the involvement of potential strain-dependent responses that require further studying. Additionally, other triterpenoids reported to inhibit HMGCR activity, like cucurbitacin E and beta boswellic acid (Ndlovu et al. 2023), showed strong strain-specific inhibition patterns amongst AOA; they were particularly active on *N. viennensis* but had minimal or no effect on *N. sinensis*. These differences across strains may arise from genomic variation in stress response and metal homeostasis pathways (Reyes et al. 2020; Glass and Orphan 2012), including copper-transporting proteins, chaperones, or efflux systems that directly influence AMO activity. Other triterpenoids, such as echinocystic acid, inhibited both AOA strains despite not being reported as HMGCR inhibitors (Han et al. 2014). Taken together, these findings suggest that HMGCR inhibition may contribute to AOA sensitivity; however, additional strain-specific and archaeal-specific mechanisms are also likely involved and the proposed inhibition mechanisms should be interpreted as working hypotheses pending biochemical validation. Further research is needed to validate the proposed inhibition mechanism at biochemical level and determine whether this suppression specifically targets AOA or involves broader archaeal-selective mechanisms.

The sustained inhibitory activity of certain triterpenoids on AOA suggests their potential suitability for prolonged suppression of nitrification in agricultural soils where AOA are the major drivers of nitrification (Leininger et al. 2006). However, the observed activity is based on *in vitro* measurements that may vary considerably under soil conditions due to biotic and abiotic interactions influencing compound behavior, persistence, and bioavailability (Zhalnina et al. 2012). In this context, recent evidence demonstrates that plant-derived triterpenoids can accumulate in the rhizosphere at measurable levels. For example, cucurbitacin B was detected at approximately 69 nmol g^−1^ dry soil in the rhizosphere of melon (Zhong et al. 2022), indicating that localized micromolar-range concentrations may occur in close proximity to roots. Comparable data are not yet available for the triterpenoids tested here and bulk soil concentrations are likely lower than those tested *in vitro*, while compound persistence, sorption, and microbial degradation may further modulate bioavailability. Nevertheless, our findings support the ecological plausibility that nitrifying microorganisms in the rhizosphere may be exposed to biologically relevant concentrations. This was clearly shown for root-exuded specialized triterpenes in *Arabidopsis thaliana*, which act as selective chemical mediators that not only shape the composition and diversity of the root microbiome at the community level but also directly modulate the growth and metabolic activity of individual bacterial strains, including serving as substrates for specific microbes (Huang et al. 2019). Further soil studies are needed to determine the true concentration levels of these compounds in soil and verify their inhibitory activity, persistence, and limited off-target effects on the soil microbiota.

## Conclusions

This study provides the first experimental evidence for the intrinsic inhibitory activity of diverse plant-derived triterpenoids on a range of ecophysiologically and taxonomically distinct soil-derived AOA and AOB. A key finding was the strong inhibitory activity of a subset of triterpenoids against both AOA strains, while only marginal or no activity was observed against AOB strains, highlighting a marked selectivity toward AOA. We hypothesize that this sensitivity may involve interference with archaeal membrane-associated processes, potentially including HMGCR inhibition; however, this interpretation remains speculative, as no direct biochemical evidence was obtained. Alternative mechanisms, such as membrane perturbation or altered permeability of archaeal lipid membranes to hydrophobic triterpenoids, cannot be rulled out. Future studies would clarify the biochemical basis of AOA sensitivity to triterpenoids and assess the environmental relevance and soil behavior of these compounds to determine their realistic potential as BNIs. Although, *in silico* modeling yielded limited insight into structural alerts for NI activity, it remains a promising strategy for early bioactive compound discovery, particularly with expanded datasets.

## Supplementary Information

Below is the link to the electronic supplementary material.

ESM 1 (PDF 1.42 MB)

## Data Availability

The datasets generated and/or analysed during the current study are available from the corresponding author on reasonable request.
