# Supplementary material for: The potential of plant-derived triterpenoids as biological nitrification inhibitors
Source: Appl Microbiol Biotechnol. 2026 Mar 17;110:107. doi: 10.1007/s00253-026-13776-3 (PMC12999751; doi:10.1007/s00253-026-13776-3)
Supplement: Supplementary file 1 [file 253_2026_13776_MOESM1_ESM.pdf]

# The potential of plant-derived triterpenoids as biological nitrification inhibitors

**Hugo Ribeiro[1], Evangelia S. Papadopoulou[2*], Kunyang Zhang[3,4], Alexandros E. Kanellopoulos[1], Kalliope K. Papadopoulou[1], Dimitrios G. Karpouzas[1]**

[1]Laboratory of Plant and Environmental Biotechnology, Department of Biochemistry and Biotechnology, University of Thessaly, Larissa, Greece

[2]Laboratory of Environmental Microbiology and Virology, Department of Environmental Sciences, University of Thessaly, Larissa, Greece

[3] Eawag, Department of Environmental Chemistry, Überlandstrasse 133, 8600 Dübendorf, Switzerland,

[4] University of Zürich, Department of Chemistry, Winterthurerstrasse 190, 8057 Zürich, Switzerland

*Corresponding author

Evangelia S. Papadopoulou

University of Thessaly

Department of Environmental Sciences

Gaiopolis, 41500 Larissa, Greece

Tel. +30-2410-684245,

Email. evapapadopoulou@uth.gr

## Supplemental Tables

**Supplemental Table S1** Ammonia oxidation inhibition (AOI %) values for known synthetic nitrification inhibitors (SNIs: ethoxyquin and DMPP), biological nitrification inhibitors (BNIs: MHPP, sakuranetin) and the tested triterpenoids at two concentration levels. Inhibition was assessed in ammonia-oxidizing archaea (AOA: *Nitrososphaera viennensis* and *Nitrosotalea sinensis*) and ammonia-oxidizing bacteria (AOB: *Nitrosospira multiformis* and *Nitrosomonas ureae*) at two time points: (i) at the end of the exponential growth phase (transition to stationary), to evaluate inhibition relative to the controls, and (ii) at the end of incubation (late stationary phase), to assess potential recovery or persistent inhibition. Transition phase occurred at day 10.0 for *N. sinensis*, day 10.5 for *N. viennensis*, and day 4.0 for *N. multiformis* and *N. ureae*; end of incubation was at day 23.0 for *N. sinensis*, day 21.0 for *N. viennensis*, and day 10.0 for *N. multiformis* and *N. ureae*. Statistical significance of t-tests versus the negative DMSO control is indicated as follows: * $p < 0.05$; ** $p < 0.01$; and *** $p < 0.001$.

| Compound Group | Compound | Concentration (mg $L^{-1}$) | Stage of activity | Ammonia-oxidizing bacteria | | Ammonia-oxidizing archaea | |
|---|---|---|---|---|---|---|---|
| | | | | *Nitrosospira multiformis* | *Nitrosomonas ureae* | *Nitrososphaera viennensis* | *Nitrosotalea sinensis* |
| **AOA-specific NIs** | Ethoxyquin (AOA-specific SNI) | 20 | Transitional | - | - | 102% (***) | 117% (***) |
| | | | End | - | - | 101% (***) | 148% (***) |
| | | 5 | Transitional | - | - | 101% (***) | 110% (***) |
| | | | End | - | - | 100% (***) | 134% (***) |
| | Sakuranetin (AOA-specific BNI) | 20 | Transitional | - | - | 96.7% (***) | 100% (***) |
| | | | End | - | - | 96.3% (***) | 111% (***) |
| | | 5 | Transitional | - | - | 55.8% (***) | 101% (***) |
| | | | End | - | - | 16.3% (**) | 98.5% (*) |
| **AOB-specific NIs** | DMPP (AOB-specific SNI) | 20 | Transitional | 101% (***) | 102% (***) | - | - |
| | | | End | 99.5% (***) | 100% (***) | - | - |
| | | 5 | Transitional | 97.6% (***) | 99.3% (***) | - | - |
| | | | End | 98.1% (***) | 97.8% (***) | - | - |
| | MHPP (AOB-specific BNI) | 100 | Transitional | 97.6% (***) | 102% (***) | - | - |
| | | | End | 97.9% (***) | 99.5% (***) | - | - |
| | | 20 | Transitional | 46.3% (***) | 72.3% (***) | - | - |
| | | | End | -0.6% | 3.7% | - | - |
| | | 5 | Transitional | 19.3% (*) | 32.9% (*) | - | - |
| | | | End | 0.0% | 4.6% | - | - |
| **Ursane-type triterpenoids** | 11-Keto-beta boswellic acid | 20 | Transitional | - 0.5% | -4.0% | 97.7% (***) | 94.0% (***) |
| | | | End | -8.9% | -16.7% (**) | 98.9% (***) | 105% (***) |
| | | 5 | Transitional | 0.8% | -7.3% | 61.5% (***) | 95.4% (***) |
| | | | End | -11.9% (*) | -11.0% (*) | 20.8% (*) | 105% (***) |
| | 3-O-acetyl-11-ketobeta boswellic acid | 20 | Transitional | -4.6% | 19.3% | 98.7% (***) | 94.2% (***) |
| | | | End | -10.1% (*) | -15.1% (*) | 98.0% (***) | 104% (***) |
| | | 5 | Transitional | 4.8% | -5.5% | 99.4% (***) | 96.2% (***) |
| | | | End | -9.5% | -24.9% (*) | 99.8% (***) | 107% (***) |
| | 3-O-acetyl-beta boswellic acid | 20 | Transitional | -3.0% | -12.7% | -2.9% | -1.1% |
| | | | End | -4.5% | -12.4% | 1.9% | -18.9% (*) |
| | | 5 | Transitional | 0.8% | -15.6% (**) | 0.4% | -0.7% |
| | | | End | -1.7% | -10.9% (*) | -3.5% | -20.0% |
| | 3-O-acetyl-alpha boswellic acid | 20 | Transitional | -4.8% | 0.7% | -2.63% | -3.3% |
| | | | End | -5.2% | -12.1% | 1.2% | -17.3% (*) |
| | | 5 | Transitional | -4.6% | -4.4% | -5.0% | -13.4% |
| | | | End | -4.1% | -10.7% | 0.4% | -15.0% (*) |
| | | 20 | Transitional | 1.9% | 4.4% | 93.7% (***) | -2.2% |

| | | | | | | | |
|---|---|---|---|---|---|---|---|
| | | | End | -0.6% | -12.7% (*) | 93.7% (***) | -3.5% |
| | Beta boswellic acid | 5 | Transitional | 5.4% | -5.1% | 93.3% (***) | 3.7% |
| | | | End | 0.4% | -13.4% | 93.4% (***) | 1.8% |
| | Asiatic acid | 20 | Transitional | -3.2% | 8.1% | 65.7% (***) | 100% (***) |
| | | | End | 0.7% | -0.5% | 61.7% (***) | 111% (***) |
| | | 5 | Transitional | 7.0% | 0.6% | -3.5% | 97.9% (***) |
| | | | End | -0.4% | 0.8% | 3.0% | 109% (***) |
| | Madecassoside | 20 | Transitional | -3.3% | 16.9% (*) | -3.9% | 4.6% |
| | | | End | -5.5% | -5.6% | 3.6% | 1.9% |
| | | 5 | Transitional | 5.2% (*) | 4.6% | -4.6% | 2.3% |
| | | | End | 3.36% | -6.7% | 0.9% | -4.7% |
| | Ursolic acid | 20 | Transitional | 27.7% (**) | 1.4% | 99.8% (***) | 74.3% (**) |
| | | | End | -7.2% (*) | -5.2% | 99.8% (***) | 79.4% (**) |
| | | 5 | Transitional | 17.0% | 5.5% | 99.1% (***) | 70.3% (*) |
| | | | End | -8.8% (*) | -5.7% | 99.2% (***) | 37.6% |
| **Oleanane-type triterpenoids** | Oleanolic acid | 20 | Transitional | 1.3% | -4.3% | 100% (***) | 8.16% |
| | | | End | -10.7% | -3.3% | 101% (***) | -7.9% |
| | | 5 | Transitional | -0.7% | -4.9% | 99.9% (***) | 48.5% (*) |
| | | | End | -12.1% | -4.9% | 100% (***) | -21.3% |
| | Echinocystic acid | 20 | Transitional | -1.2% | -8.1% (*) | 87.4% (***) | 88.5% (***) |
| | | | End | -10.7% | -5.9% | 76.3% (***) | 93.7% (***) |
| | | 5 | Transitional | -1.6% | -7.0% | 49.3% (***) | 74.9% (*) |
| | | | End | -8.1% | -5.4% | 5.7% | 35.6% |
| | Echinocystic acid-3-O-glucoside | 20 | Transitional | -1.2% | -12.7% (*) | 28.8% (*) | 94.4% (***) |
| | | | End | -7.8% | -6.2% | 4.6% | 106% (***) |
| | | 5 | Transitional | -4.1% (**) | -11.3% | 30.5% (**) | 86.0% (***) |
| | | | End | -8.9% | -10.4% | 2.6% | 94.0% (***) |
| | Chrysanthellin A | 20 | Transitional | -3.7% (*) | -13.5% (**) | 1.1% | 7.0% |
| | | | End | -12.6% (*) | -8.3% (*) | -1.7% | -3.9% |
| | | 5 | Transitional | -7.7% (*) | -18.4% (**) | -6.9% (*) | 1.0% |
| | | | End | -15.6% (*) | -4.1% | -0.1% | -12.8% (*) |
| | Chrysanthellin B | 20 | Transitional | -4.0% | -16.6% (*) | 6.5% | 10.1% |
| | | | End | -16.5% (*) | -8.6% (*) | -2.9% | 2.2% |
| | | 5 | Transitional | -0.9% | -12.2% (**) | -4.6% | 3.6% |
| | | | End | -14.4% (***) | -9.6% | 2.9% | -10.7% |
| | Betulin | 5 | Transitional | 5.9% (*) | -3.2% | -2.9% | 30.5% (***) |

| | | | | | | | |
|---|---|---|---|---|---|---|---|
| **Lupane-type triterpenoids** | | | End | -4.2% (*) | -4.1% | 4.7% | 19.7% (*) |
| | | 2.5 | Transitional | 8.3% (*) | -2.2% | -2.5% | 34.7% (*) |
| | | | End | -5.7% (*) | -4.1% | 8.4% | 9.7% |
| | Betulinic acid | 20 | Transitional | 6.6% | -2.1% | -3.4% | 13.1% (**) |
| | | | End | -7.8% (*) | -5.2% (*) | 3.1% | 0.2% |
| | | 5 | Transitional | 0.6% | -4.8% | -2.0% | 6.4% |
| | | | End | -7.5% (*) | -7.1% (*) | 1.8% | -4.3% |
| **Cucurbitane-type triterpenoids** | Cucurbitacin D | 20 | Transitional | -1.1% | -3.7% | -5.0% | 11.0% |
| | | | End | -12.7% (*) | -6.5% (*) | 1.9% | -7.3% |
| | | 5 | Transitional | 1.5% | 4.2% | 0.4% | 6.3% (*) |
| | | | End | -6.8% (**) | -4.6% (*) | 8.1% | -21.0% (*) |
| | Cucurbitacin E | 20 | Transitional | - 0.9% | - 8.0% | 66.8% (***) | 14.6% (**) |
| | | | End | -12.9% (**) | -8.7% | 7.7% (*) | -9.0% |
| | | 5 | Transitional | -0.1% | 4.5% | 4.1% | 6.6% (*) |
| | | | End | -8.9% (**) | -5.8% | 5.7% | -21.3% (*) |
| | Cucurbitacin I | 20 | Transitional | -1.8% | -13.3% (**) | 17.1% (**) | 13.3% |
| | | | End | -12.0% | -6.7% (*) | 6.13% (*) | 0.1% |
| | | 5 | Transitional | -1.0% | -7.8% (*) | 17.4% | 11.1% (**) |
| | | | End | -9.0% | -5.0% | -0.9% | -16.7% (*) |

**Supplemental Table S2** *In silico* predictions using a model trained exclusively on AOB data (Zhang et al. 2025). The model predicted whether the half maximal effective concentration ($EC_{50}$) of each compound (known nitrification inhibitors and triterpenoids) was lower (classified as positive) or higher (classified as negative) than 80 mg $L^{-1}$, according to the criteria defined in that study.

| | Compounds | Prediction on AOB | Prediction on AOA |
|---|---|---|---|
| AOB – specific NIs | 3,4-dimethylpyrazole phosphate (DMPP) | Positive | Not tested |
| | Methyl 3-(4-hydroxyphenyl)propionate (MHPP) | Positive | Not tested |
| AOA – specific NIs | Ethoxyquin | Not tested | Positive |
| | Sakuranetin | Not tested | Negative |
| Triterpenoids | 11-keto-beta boswellic acid | Negative | Negative |
| | 3-O-Acetyl-11-ketobeta Boswellic Acid | Negative | Negative |
| | 3-O-Acetyl-beta Boswellic Acid | Negative | Negative |
| | 3-O-Acetyl-alpha Boswellic Acid | Negative | Negative |
| | Beta Boswellic Acid | Negative | Negative |
| | Asiatic Acid | Negative | Negative |
| | Madecassoside | Negative | Negative |
| | Betulin | Negative | Negative |
| | Betulinic Acid | Negative | Negative |
| | Chrysanthellin A | Negative | Negative |
| | Chrysanthellin B | Negative | Negative |
| | Cucurbitacin D | Negative | Negative |
| | Cucurbitacin E | Negative | Negative |
| | Cucurbitacin I | Negative | Negative |
| | Echinocystic Acid | Negative | Negative |
| | Echinocystic Acid-3-O-Glucoside | Negative | Negative |
| | Oleanolic Acid | Negative | Negative |
| | Ursolic Acid | Negative | Negative |

# Supplementary Figures

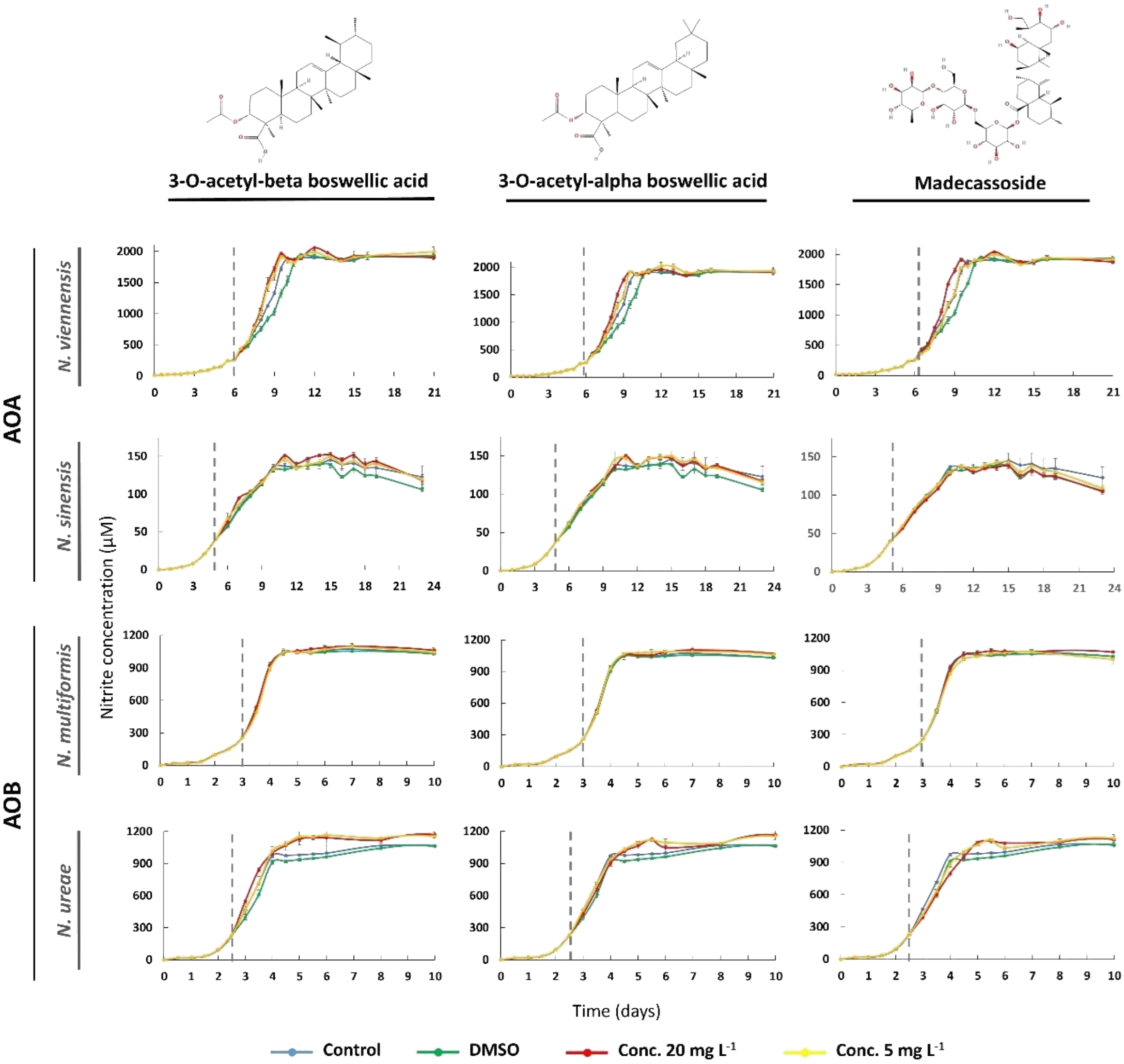


**Supplementary Fig. S1** Effect of a subset of ursane-type triterpenoids (3-O-acetyl-beta-boswellic acid, 3-O-acetyl-alpha-boswellic acid, and madecassoside) that showed limited inhibitory activity on ammonia-oxidizing archaea (*Nitrososphaera viennensis*, *Nitrosotalea sinensis*) and ammonia-oxidizing bacteria (*Nitrosospira multiformis*, *Nitrosomonas ureae*). Compounds were tested at 5 and 20 mg $L^{-1}$. Activity was assessed by monitoring nitrite production. Error bars represent the standard error of the mean of triplicate cultures. The vertical dashed line indicates the time at which the compounds were added to the cultures.

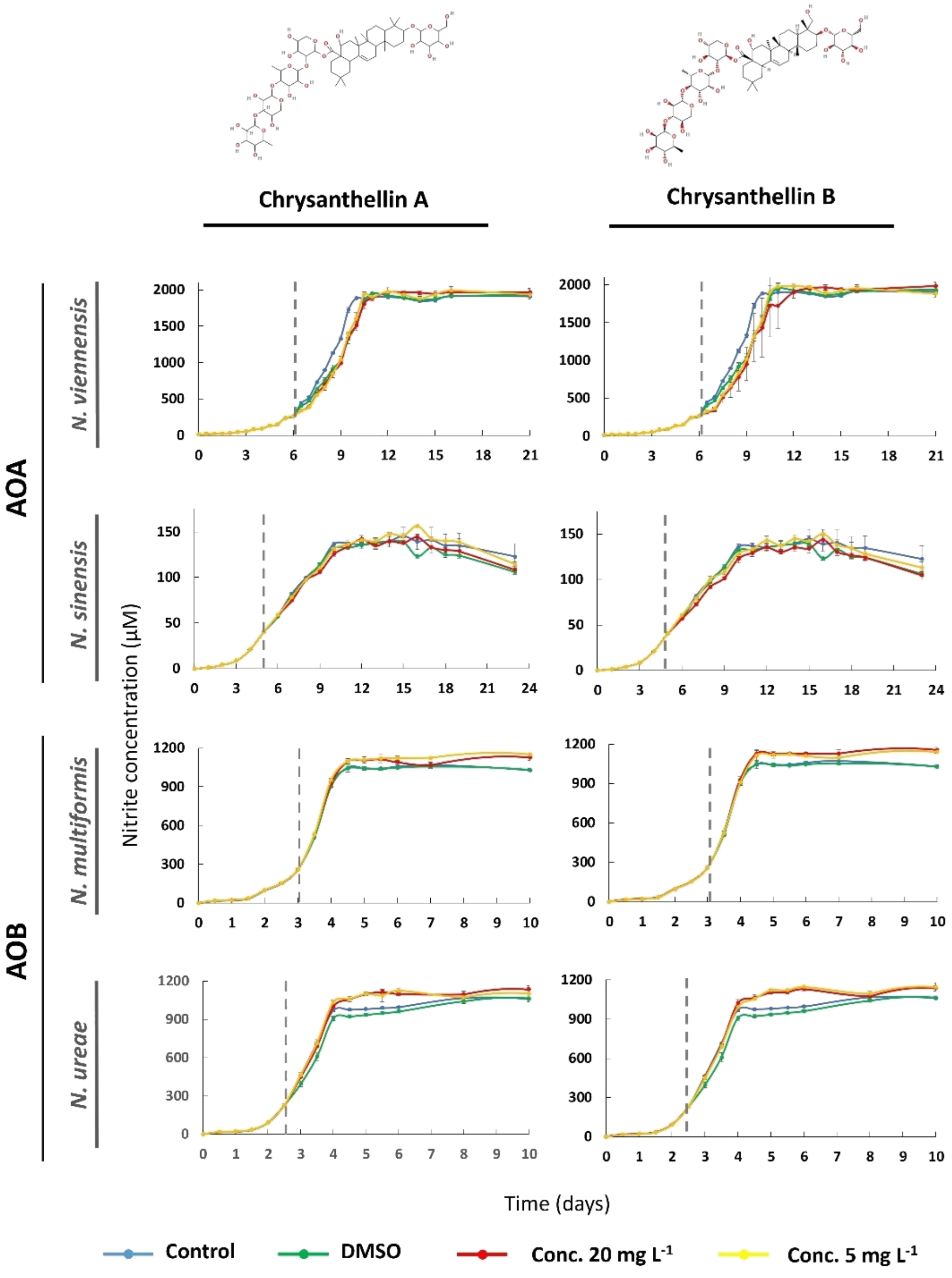


**Supplementary Fig. S2** Effect of a subset of oleanane-type triterpenoids (chrysanthellin A and chrysanthellin B) that showed limited inhibitory activity on ammonia-oxidizing archaea (*Nitrososphaera viennensis*, *Nitrosotalea sinensis*) and ammonia-oxidizing bacteria (*Nitrosospira multiformis*, *Nitrosomonas ureae*). Compounds were tested at 5 and 20 mg $L^{-1}$. Activity was assessed by monitoring nitrite production. Error bars represent the standard error of the mean of triplicate cultures. The vertical dashed line indicates the time at which the compounds were added to the cultures.

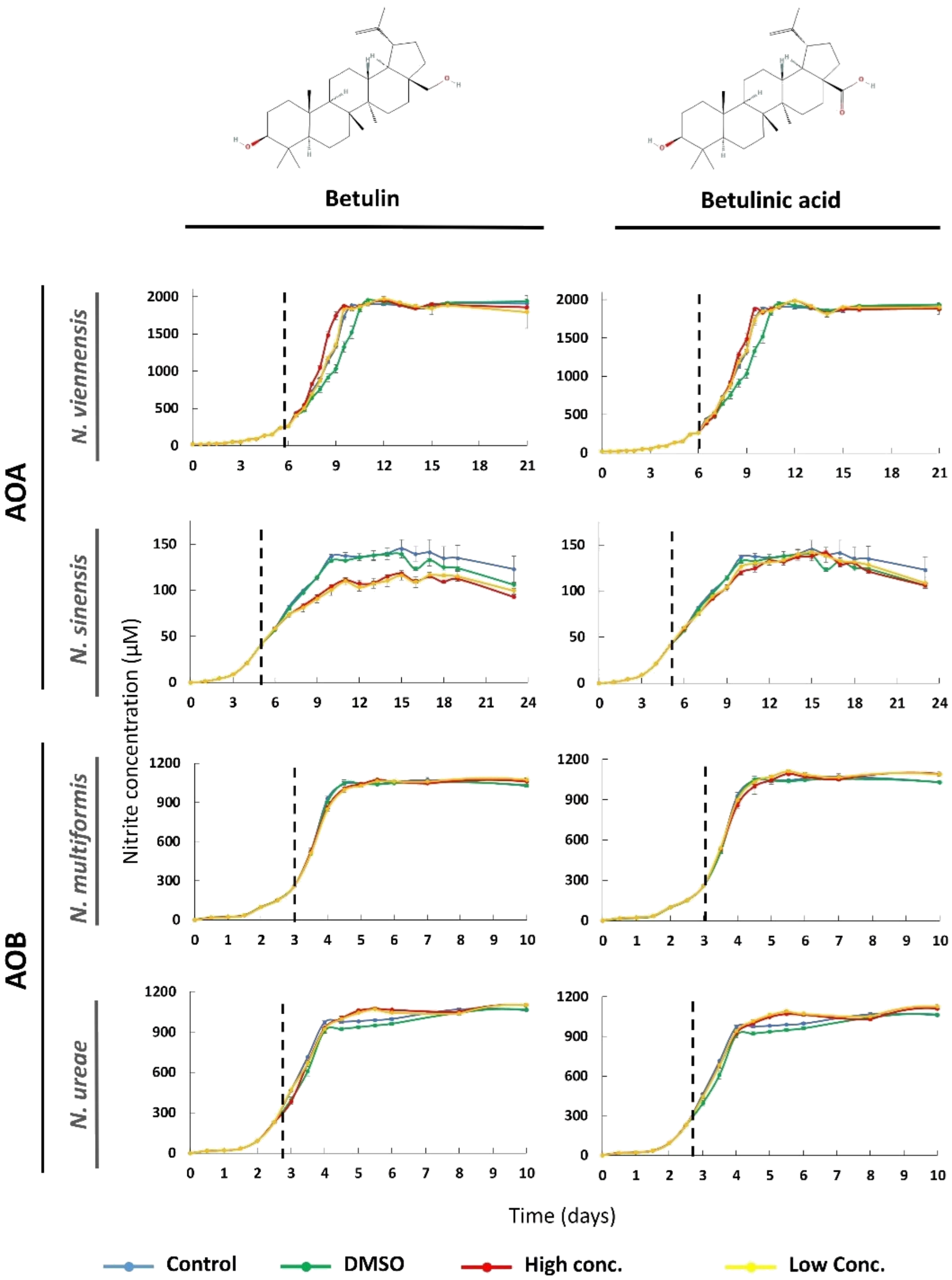


**Supplementary Fig. S3** Effect of lupane-type triterpenoids (betulin, tested at 2.5 and 5 mg $L^{-1}$, and betulinic acid, tested at 5 and 20 mg $L^{-1}$) on ammonia-oxidizing archaea (*Nitrososphaera viennensis* and *Nitrosotalea sinensis*) and ammonia-oxidizing bacteria (*Nitrosospira multiformis* and *Nitrosomonas ureae*). Activity was assessed by monitoring nitrite production. Error bars represent the standard error of the mean of triplicate cultures. The vertical dashed line indicates the time at which the compounds were added to the cultures.

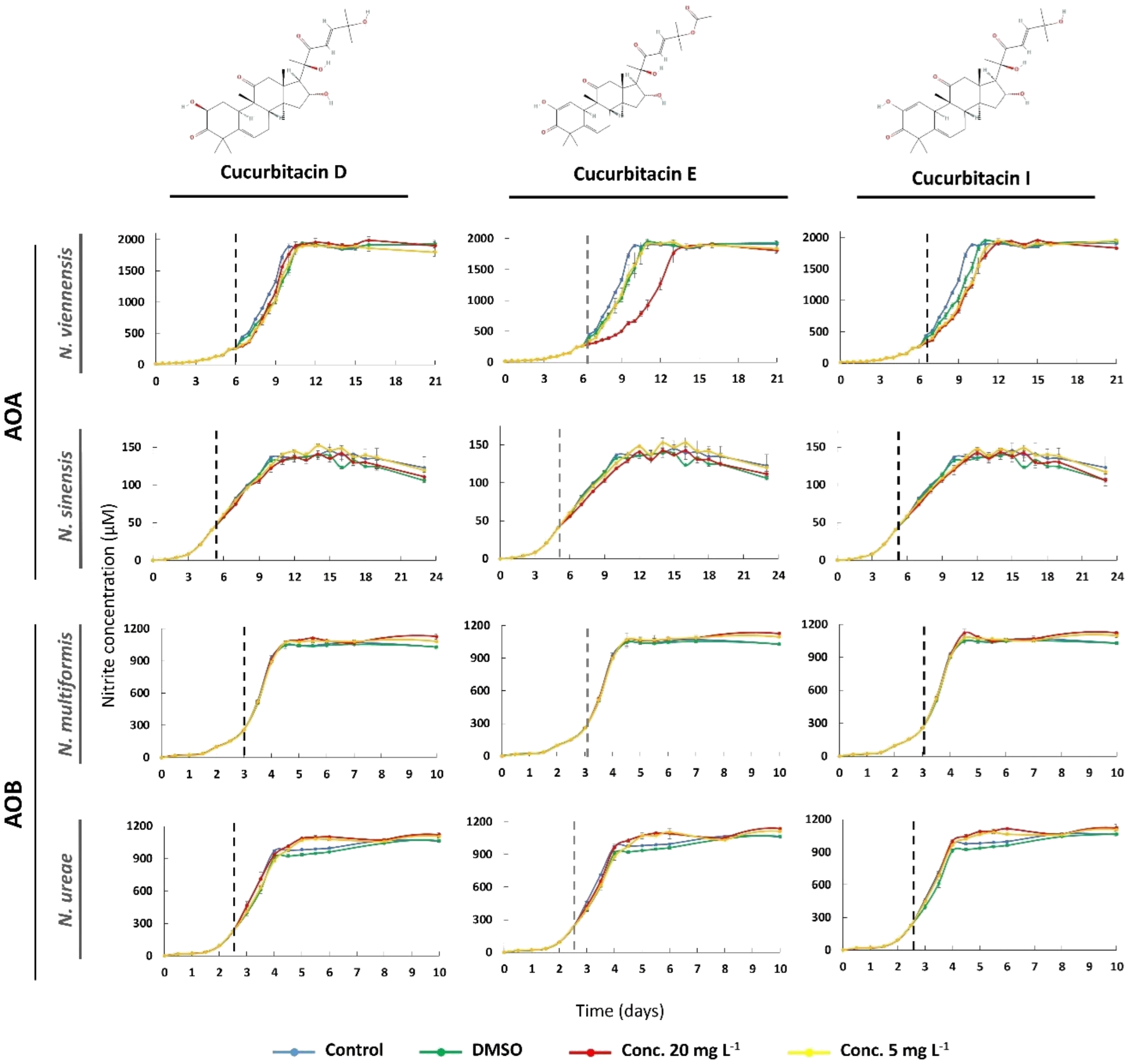


**Supplementary Fig. S4** Effect of cucurbitane-type triterpenoids (cucurbitacin D, E and cucurbitacin I) on ammonia-oxidizing archaea (*Nitrososphaera viennensis* and *Nitrosotalea sinensis*) and ammonia-oxidizing bacteria (*Nitrosospira multiformis* and *Nitrosomonas ureae*). Compounds were tested at 5 and 20 mg $L^{-1}$. Activity was assessed by monitoring nitrite production. Error bars represent the standard error of the mean of triplicate cultures. The vertical dashed line indicates the time at which the compounds were added to the cultures.

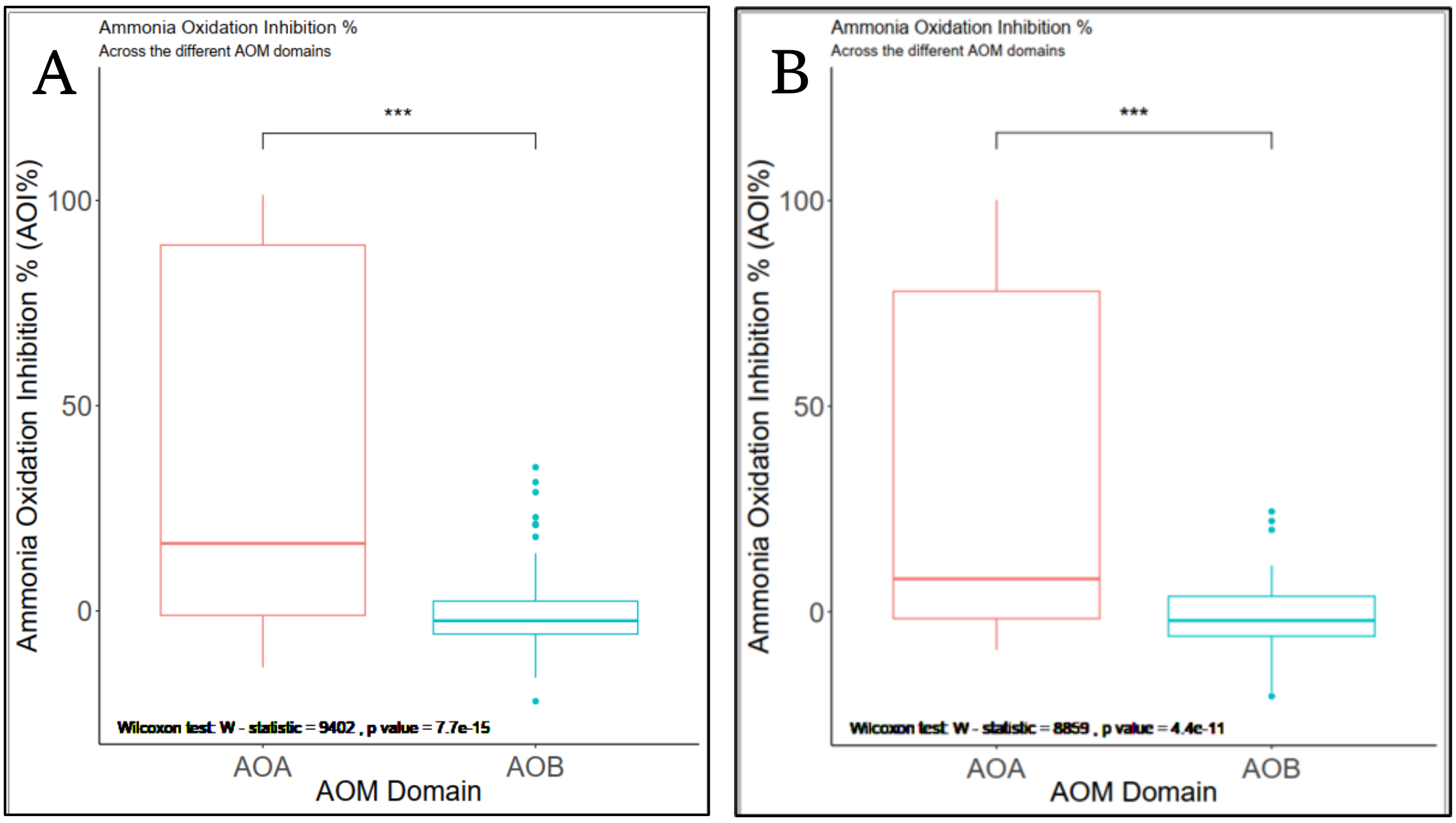


**Supplementary Fig. S5** Comparison of mean ammonia oxidation inhibition (AOI, %) values for (A) high (20 mg $L^{-1}$) and (B) low (5 mg $L^{-1}$) concentrations of triterpenoids on ammonia-oxidizing archaea (AOA: *Nitrososphaera viennensis* and *Nitrosotalea sinensis*) and ammonia-oxidizing bacteria (AOB: *Nitrosospira multiformis* and *Nitrosomonas ureae*). Asterisks (***) indicate statistical significance ($p < 0.001$). The Wilcoxon test F-statistic and $p$-value are provided in the bottom left corner.

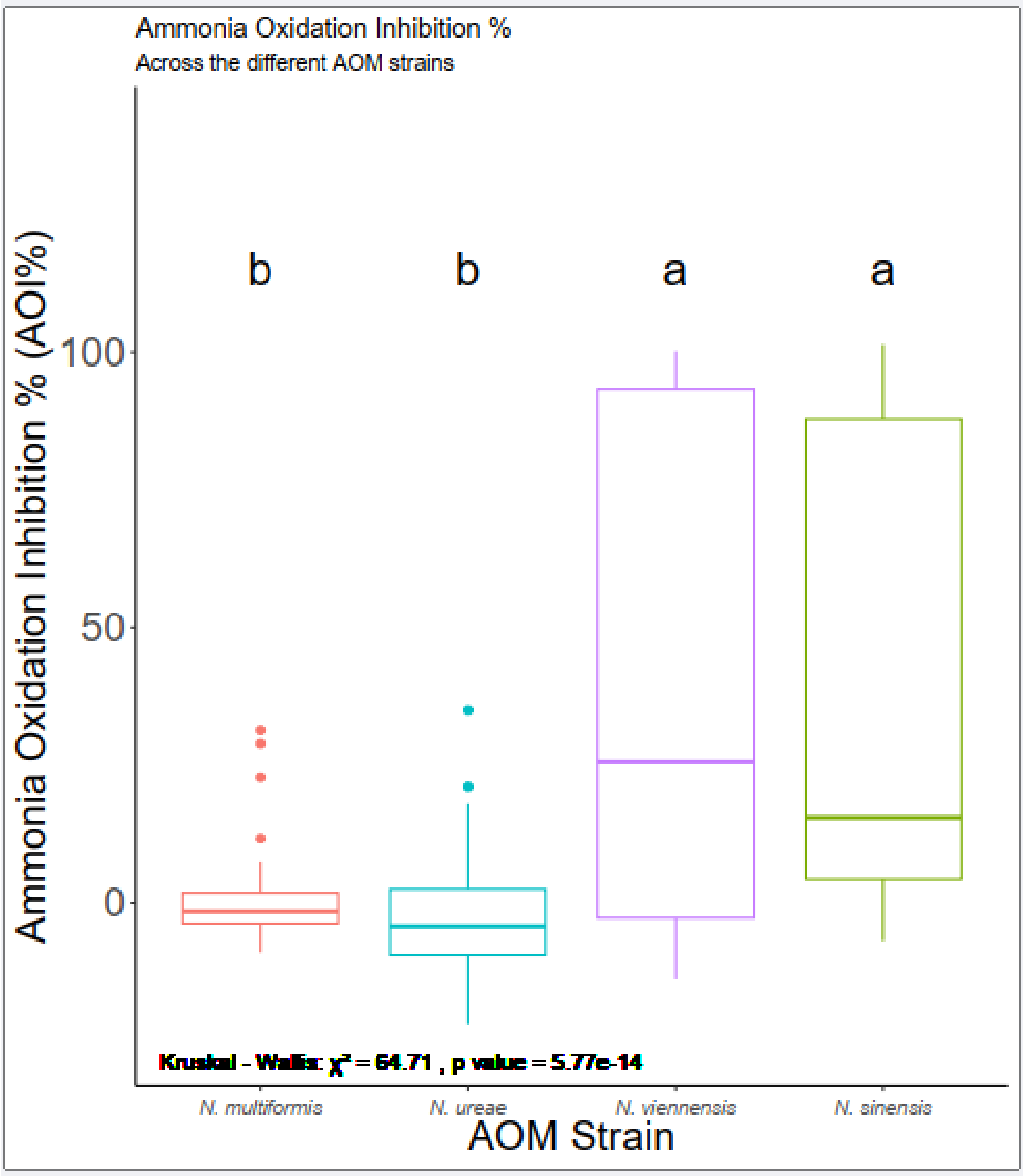


**Supplementary Fig. S6** Comparison of mean ammonia oxidation inhibition (AOI, %) values for the high concentration (20 mg $L^{-1}$) of the tested triterpenoids on the different ammonia-oxidizing archaeal (AOA: *Nitrososphaera viennensis* and *Nitrosotalea sinensis*) and ammonia-oxidizing bacterial strains (AOB: *Nitrosospira multiformis* and *Nitrosomonas ureae*). Boxes denoted by different lowercase letters indicate statistical significance and grouping according to Kruskal – Wallis Dunn's post – hoc test ($p < 0.05$).
