# Supplementary material for: The potential of plant-derived triterpenoids as biological nitrification inhibitors
Source: Appl Microbiol Biotechnol. 2026 Mar 17;110(1):107. doi: 10.1007/s00253-026-13776-3 (PMC12999751; doi:10.1007/s00253-026-13776-3)
Supplement: Supplementary file 1 — (PDF 1.42 MB) [file 253_2026_13776_MOESM1_ESM.pdf]

The potential of plant-derived triterpenoids as biological nitrification inhibitors

Hugo Ribeiro[1], Evangelia S. Papadopoulou[2*], Kunyang Zhang[3,4], Alexandros E. Kanellopoulos[1], Kalliope K. Papadopoulou[1], Dimitrios G. Karpouzas[1]

[1]Laboratory of Plant and Environmental Biotechnology, Department of Biochemistry and Biotechnology, University of Thessaly, Larissa, Greece

[2]Laboratory of Environmental Microbiology and Virology, Department of Environmental Sciences, University of Thessaly, Larissa, Greece

[3] Eawag, Department of Environmental Chemistry, Überlandstrasse 133, 8600 Dübendorf, Switzerland,

[4] University of Zürich, Department of Chemistry, Winterthurerstrasse 190, 8057 Zürich, Switzerland

*Corresponding author

Evangelia S. Papadopoulou

University of Thessaly

Department of Environmental Sciences

Gaiopolis, 41500 Larissa, Greece

Tel. +30-2410-684245,

Email. evapapadopoulou@uth.gr

Supplemental Tables

Supplemental Table S1 Ammonia oxidation inhibition (AOI %) values for known synthetic nitrification inhibitors (SNIs: ethoxyquin and DMPP), biological nitrification inhibitors (BNIs: MHPP, sakuranetin) and the tested triterpenoids at two concentration levels. Inhibition was assessed in ammonia-oxidizing archaea (AOA: *Nitrososphaera viennensis* and *Nitrosotalea sinensis*) and ammonia-oxidizing bacteria (AOB: *Nitrosospira multiformis* and *Nitrosomonas ureae*) at two time points: (i) at the end of the exponential growth phase (transition to stationary), to evaluate inhibition relative to the controls, and (ii) at the end of incubation (late stationary phase), to assess potential recovery or persistent inhibition. Transition phase occurred at day 10.0 for *N. sinensis*, day 10.5 for *N. viennensis*, and day 4.0 for *N. multiformis* and *N. ureae*; end of incubation was at day 23.0 for *N. sinensis*, day 21.0 for *N. viennensis*, and day 10.0 for *N. multiformis* and *N. ureae*. Statistical significance of t-tests versus the negative DMSO control is indicated as follows: * $p < 0.05$; ** $p < 0.01$; and *** $p < 0.001$.

Compound Group	Compound	Concentration (mg L^{-1})	Stage of activity	Ammonia-oxidizing bacteria		Ammonia-oxidizing archaea	
				Nitrosospira multiformis	*Nitrosomonas ureae*	*Nitrososphaera viennensis*	*Nitrosotalea sinensis*
AOA-specific NIs	Ethoxyquin (AOA-specific SNI)	20	Transitional	-	-	102% (***)	117% (***)
			End	-	-	101% (***)	148% (***)
		5	Transitional	-	-	101% (***)	110% (***)
			End	-	-	100% (***)	134% (***)
	Sakuranetin (AOA-specific BNI)	20	Transitional	-	-	96.7% (***)	100% (***)
			End	-	-	96.3% (***)	111% (***)
		5	Transitional	-	-	55.8% (***)	101% (***)
			End	-	-	16.3% (**)	98.5% (*)
AOB-specific NIs	DMPP (AOB-specific SNI)	20	Transitional	101% (***)	102% (***)	-	-
			End	99.5% (***)	100% (***)	-	-
		5	Transitional	97.6% (***)	99.3% (***)	-	-
			End	98.1% (***)	97.8% (***)	-	-
	MHPP (AOB-specific BNI)	100	Transitional	97.6% (***)	102% (***)	-	-
			End	97.9% (***)	99.5% (***)	-	-
		20	Transitional	46.3% (***)	72.3% (***)	-	-
			End	-0.6%	3.7%	-	-
		5	Transitional	19.3% (*)	32.9% (*)	-	-
			End	0.0%	4.6%	-	-
Ursane-type triterpenoids	11-Keto-beta boswellic acid	20	Transitional	- 0.5%	-4.0%	97.7% (***)	94.0% (***)
			End	-8.9%	-16.7% (**)	98.9% (***)	105% (***)
		5	Transitional	0.8%	-7.3%	61.5% (***)	95.4% (***)
			End	-11.9% (*)	-11.0% (*)	20.8% (*)	105% (***)
	3-O-acetyl-11-ketobeta boswellic acid	20	Transitional	-4.6%	19.3%	98.7% (***)	94.2% (***)
			End	-10.1% (*)	-15.1% (*)	98.0% (***)	104% (***)
		5	Transitional	4.8%	-5.5%	99.4% (***)	96.2% (***)
			End	-9.5%	-24.9% (*)	99.8% (***)	107% (***)
	3-O-acetyl-beta boswellic acid	20	Transitional	-3.0%	-12.7%	-2.9%	-1.1%
			End	-4.5%	-12.4%	1.9%	-18.9% (*)
		5	Transitional	0.8%	-15.6% (**)	0.4%	-0.7%
			End	-1.7%	-10.9% (*)	-3.5%	-20.0%
	3-O-acetyl-alpha boswellic acid	20	Transitional	-4.8%	0.7%	-2.63%	-3.3%
			End	-5.2%	-12.1%	1.2%	-17.3% (*)
		5	Transitional	-4.6%	-4.4%	-5.0%	-13.4%
			End	-4.1%	-10.7%	0.4%	-15.0% (*)
		20	Transitional	1.9%	4.4%	93.7% (***)	-2.2%

			End	-0.6%	-12.7% (*)	93.7% (***)	-3.5%
	Beta boswellic acid	5	Transitional	5.4%	-5.1%	93.3% (***)	3.7%
			End	0.4%	-13.4%	93.4% (***)	1.8%
	Asiatic acid	20	Transitional	-3.2%	8.1%	65.7% (***)	100% (***)
			End	0.7%	-0.5%	61.7% (***)	111% (***)
		5	Transitional	7.0%	0.6%	-3.5%	97.9% (***)
			End	-0.4%	0.8%	3.0%	109% (***)
	Madecassoside	20	Transitional	-3.3%	16.9% (*)	-3.9%	4.6%
			End	-5.5%	-5.6%	3.6%	1.9%
		5	Transitional	5.2% (*)	4.6%	-4.6%	2.3%
			End	3.36%	-6.7%	0.9%	-4.7%
	Ursolic acid	20	Transitional	27.7% (**)	1.4%	99.8% (***)	74.3% (**)
			End	-7.2% (*)	-5.2%	99.8% (***)	79.4% (**)
		5	Transitional	17.0%	5.5%	99.1% (***)	70.3% (*)
			End	-8.8% (*)	-5.7%	99.2% (***)	37.6%
**Oleanane-type triterpenoids**	Oleanolic acid	20	Transitional	1.3%	-4.3%	100% (***)	8.16%
			End	-10.7%	-3.3%	101% (***)	-7.9%
		5	Transitional	-0.7%	-4.9%	99.9% (***)	48.5% (*)
			End	-12.1%	-4.9%	100% (***)	-21.3%
	Echinocystic acid	20	Transitional	-1.2%	-8.1% (*)	87.4% (***)	88.5% (***)
			End	-10.7%	-5.9%	76.3% (***)	93.7% (***)
		5	Transitional	-1.6%	-7.0%	49.3% (***)	74.9% (*)
			End	-8.1%	-5.4%	5.7%	35.6%
	Echinocystic acid-3-O-glucoside	20	Transitional	-1.2%	-12.7% (*)	28.8% (*)	94.4% (***)
			End	-7.8%	-6.2%	4.6%	106% (***)
		5	Transitional	-4.1% (**)	-11.3%	30.5% (**)	86.0% (***)
			End	-8.9%	-10.4%	2.6%	94.0% (***)
	Chrysanthellin A	20	Transitional	-3.7% (*)	-13.5% (**)	1.1%	7.0%
			End	-12.6% (*)	-8.3% (*)	-1.7%	-3.9%
		5	Transitional	-7.7% (*)	-18.4% (**)	-6.9% (*)	1.0%
			End	-15.6% (*)	-4.1%	-0.1%	-12.8% (*)
	Chrysanthellin B	20	Transitional	-4.0%	-16.6% (*)	6.5%	10.1%
			End	-16.5% (*)	-8.6% (*)	-2.9%	2.2%
		5	Transitional	-0.9%	-12.2% (**)	-4.6%	3.6%
			End	-14.4% (***)	-9.6%	2.9%	-10.7%
	Betulin	5	Transitional	5.9% (*)	-3.2%	-2.9%	30.5% (***)

**Lupane-type triterpenoids**			End	-4.2% (*)	-4.1%	4.7%	19.7% (*)
		2.5	Transitional	8.3% (*)	-2.2%	-2.5%	34.7% (*)
			End	-5.7% (*)	-4.1%	8.4%	9.7%
	Betulinic acid	20	Transitional	6.6%	-2.1%	-3.4%	13.1% (**)
			End	-7.8% (*)	-5.2% (*)	3.1%	0.2%
		5	Transitional	0.6%	-4.8%	-2.0%	6.4%
			End	-7.5% (*)	-7.1% (*)	1.8%	-4.3%
**Cucurbitane-type triterpenoids**	Cucurbitacin D	20	Transitional	-1.1%	-3.7%	-5.0%	11.0%
			End	-12.7% (*)	-6.5% (*)	1.9%	-7.3%
		5	Transitional	1.5%	4.2%	0.4%	6.3% (*)
			End	-6.8% (**)	-4.6% (*)	8.1%	-21.0% (*)
	Cucurbitacin E	20	Transitional	- 0.9%	- 8.0%	66.8% (***)	14.6% (**)
			End	-12.9% (**)	-8.7%	7.7% (*)	-9.0%
		5	Transitional	-0.1%	4.5%	4.1%	6.6% (*)
			End	-8.9% (**)	-5.8%	5.7%	-21.3% (*)
	Cucurbitacin I	20	Transitional	-1.8%	-13.3% (**)	17.1% (**)	13.3%
			End	-12.0%	-6.7% (*)	6.13% (*)	0.1%
		5	Transitional	-1.0%	-7.8% (*)	17.4%	11.1% (**)
			End	-9.0%	-5.0%	-0.9%	-16.7% (*)

Supplemental Table S2 *In silico* predictions using a model trained exclusively on AOB data (Zhang et al. 2025). The model predicted whether the half maximal effective concentration (EC_{50}) of each compound (known nitrification inhibitors and triterpenoids) was lower (classified as positive) or higher (classified as negative) than 80 mg L^{-1}, according to the criteria defined in that study.

	**Compounds**	**Prediction on AOB**	**Prediction on AOA**
AOB – specific NIs	3,4-dimethylpyrazole phosphate (DMPP)	Positive	Not tested
	Methyl 3-(4-hydroxyphenyl)propionate (MHPP)	Positive	Not tested
AOA – specific NIs	Ethoxyquin	Not tested	Positive
	Sakuranetin	Not tested	Negative
Triterpenoids	11-keto-beta boswellic acid	Negative	Negative
	3-O-Acetyl-11-ketobeta Boswellic Acid	Negative	Negative
	3-O-Acetyl-beta Boswellic Acid	Negative	Negative
	3-O-Acetyl-alpha Boswellic Acid	Negative	Negative
	Beta Boswellic Acid	Negative	Negative
	Asiatic Acid	Negative	Negative
	Madecassoside	Negative	Negative
	Betulin	Negative	Negative
	Betulinic Acid	Negative	Negative
	Chrysanthellin A	Negative	Negative
	Chrysanthellin B	Negative	Negative
	Cucurbitacin D	Negative	Negative
	Cucurbitacin E	Negative	Negative
	Cucurbitacin I	Negative	Negative
	Echinocystic Acid	Negative	Negative
	Echinocystic Acid-3-O-Glucoside	Negative	Negative
	Oleanolic Acid	Negative	Negative
	Ursolic Acid	Negative	Negative

Supplementary Figures

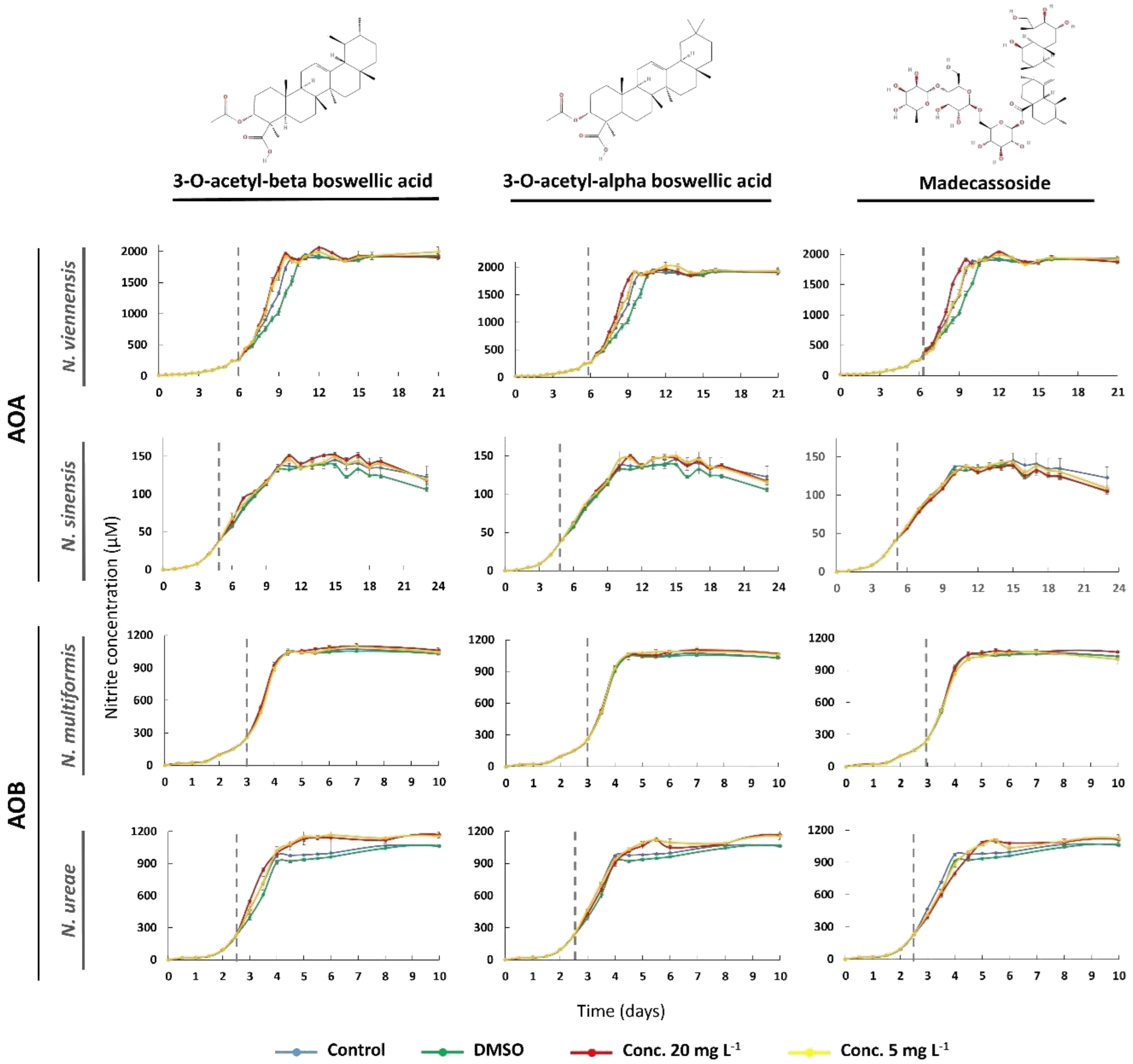

Supplementary Fig. S1 Effect of a subset of ursane-type triterpenoids (3-O-acetyl-beta-boswellic acid, 3-O-acetyl-alpha-boswellic acid, and madecassoside) that showed limited inhibitory activity on ammonia-oxidizing archaea (*Nitrososphaera viennensis*, *Nitrosotalea sinensis*) and ammonia-oxidizing bacteria (*Nitrosospira multiformis*, *Nitrosomonas ureae*). Compounds were tested at 5 and 20 mg L^{-1}. Activity was assessed by monitoring nitrite production. Error bars represent the standard error of the mean of triplicate cultures. The vertical dashed line indicates the time at which the compounds were added to the cultures.

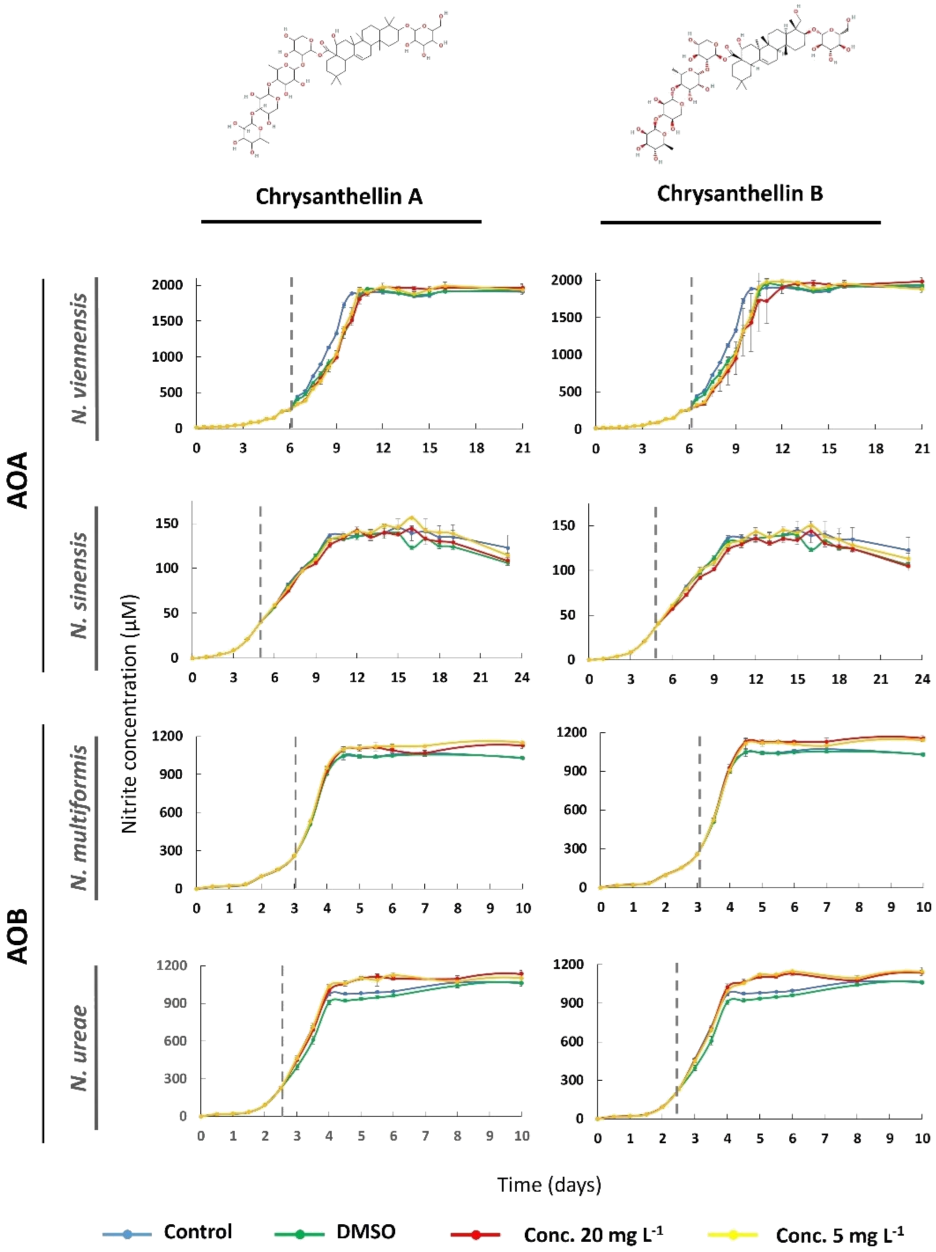

Supplementary Fig. S2 Effect of a subset of oleanane-type triterpenoids (chrysanthellin A and chrysanthellin B) that showed limited inhibitory activity on ammonia-oxidizing archaea (*Nitrososphaera viennensis*, *Nitrosotalea sinensis*) and ammonia-oxidizing bacteria (*Nitrosospira multiformis*, *Nitrosomonas ureae*). Compounds were tested at 5 and 20 mg L^{-1}. Activity was assessed by monitoring nitrite production. Error bars represent the standard error of the mean of triplicate cultures. The vertical dashed line indicates the time at which the compounds were added to the cultures.

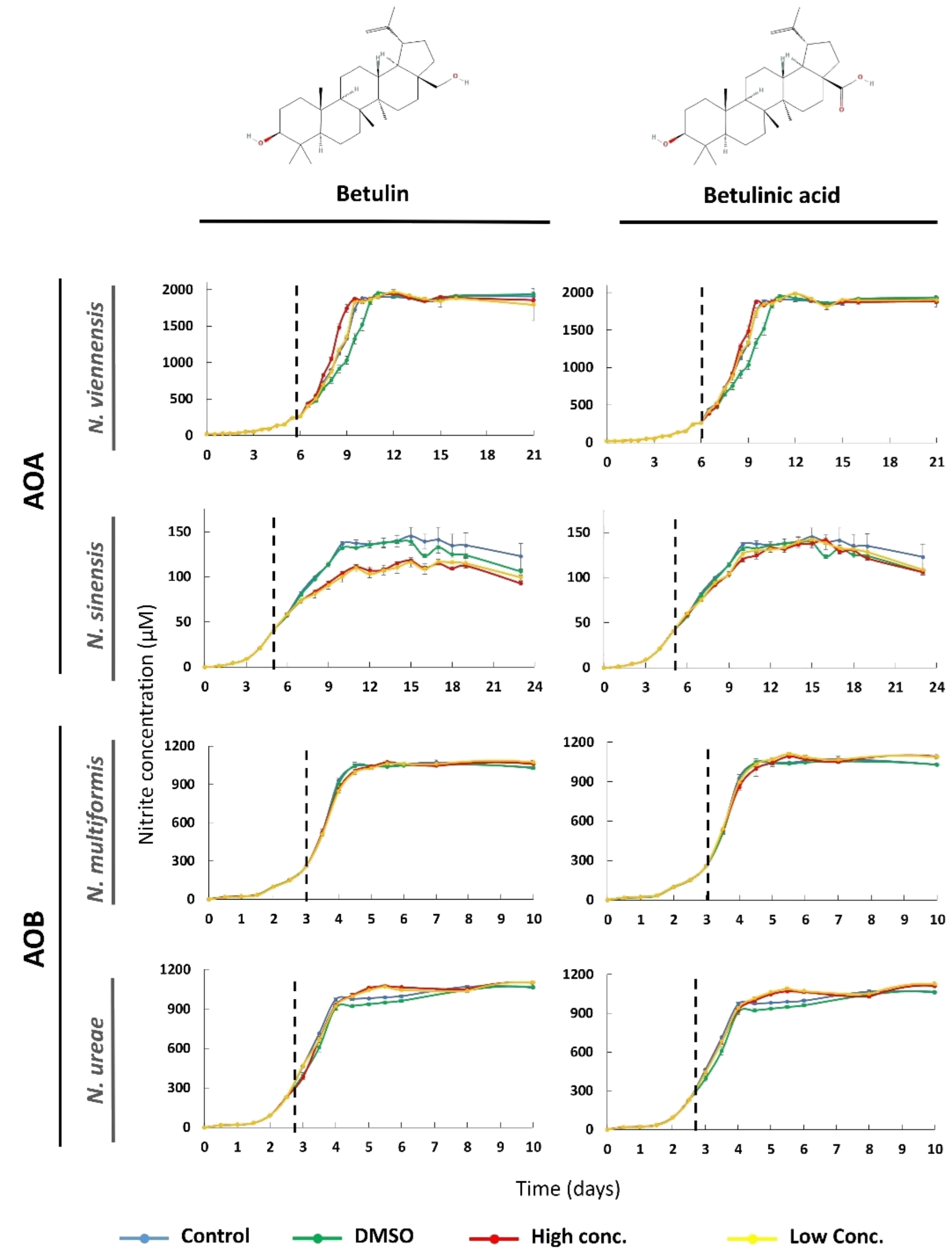

Supplementary Fig. S3 Effect of lupane-type triterpenoids (betulin, tested at 2.5 and 5 mg L^{-1}, and betulinic acid, tested at 5 and 20 mg L^{-1}) on ammonia-oxidizing archaea (*Nitrososphaera viennensis* and *Nitrosotalea sinensis*) and ammonia-oxidizing bacteria (*Nitrosospira multiformis* and *Nitrosomonas ureae*). Activity was assessed by monitoring nitrite production. Error bars represent the standard error of the mean of triplicate cultures. The vertical dashed line indicates the time at which the compounds were added to the cultures.

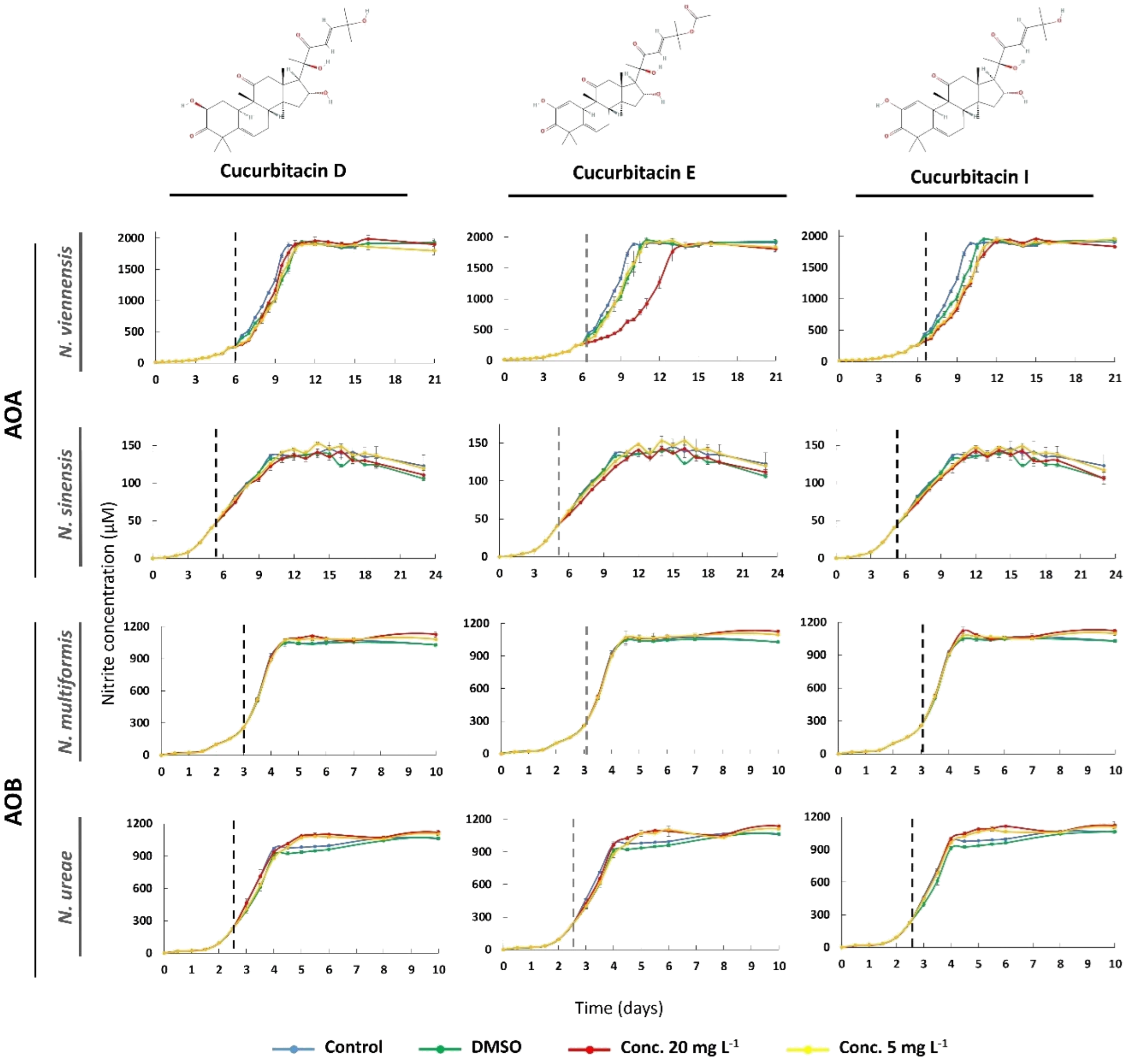

Supplementary Fig. S4 Effect of cucurbitane-type triterpenoids (cucurbitacin D, E and cucurbitacin I) on ammonia-oxidizing archaea (*Nitrososphaera viennensis* and *Nitrosotalea sinensis*) and ammonia-oxidizing bacteria (*Nitrosospira multiformis* and *Nitrosomonas ureae*). Compounds were tested at 5 and 20 mg L^{-1}. Activity was assessed by monitoring nitrite production. Error bars represent the standard error of the mean of triplicate cultures. The vertical dashed line indicates the time at which the compounds were added to the cultures.

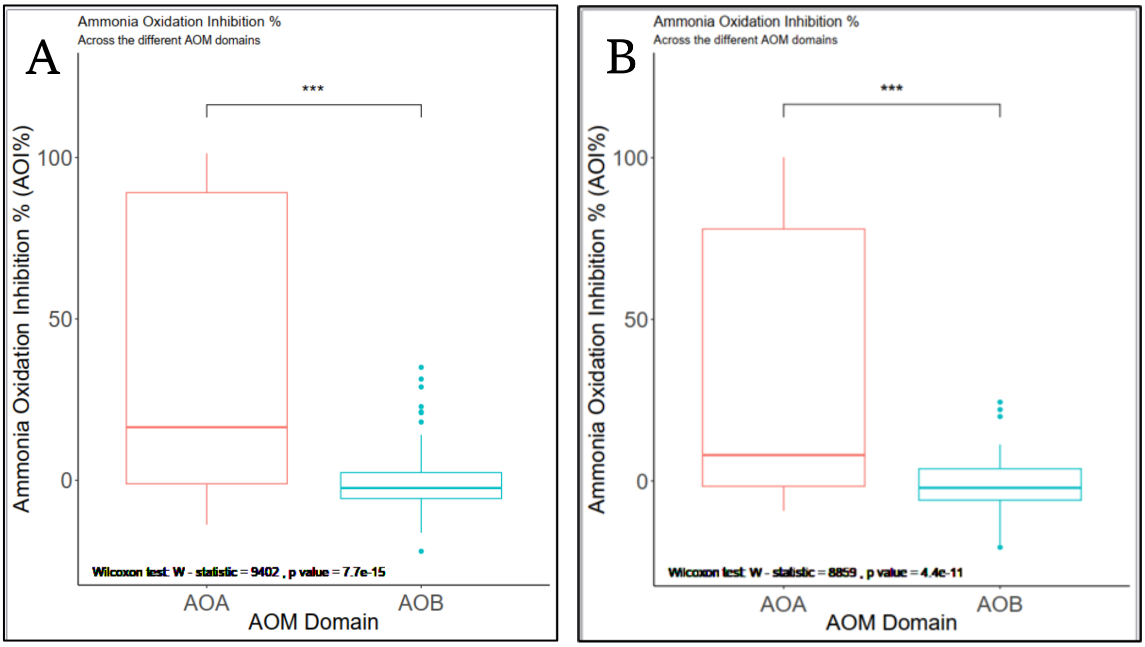

Supplementary Fig. S5 Comparison of mean ammonia oxidation inhibition (AOI, %) values for (A) high (20 mg L^{-1}) and (B) low (5 mg L^{-1}) concentrations of triterpenoids on ammonia-oxidizing archaea (AOA: *Nitrososphaera viennensis* and *Nitrosotalea sinensis*) and ammonia-oxidizing bacteria (AOB: *Nitrosospira multiformis* and *Nitrosomonas ureae*). Asterisks (***) indicate statistical significance ($p < 0.001$). The Wilcoxon test F-statistic and *p*-value are provided in the bottom left corner.

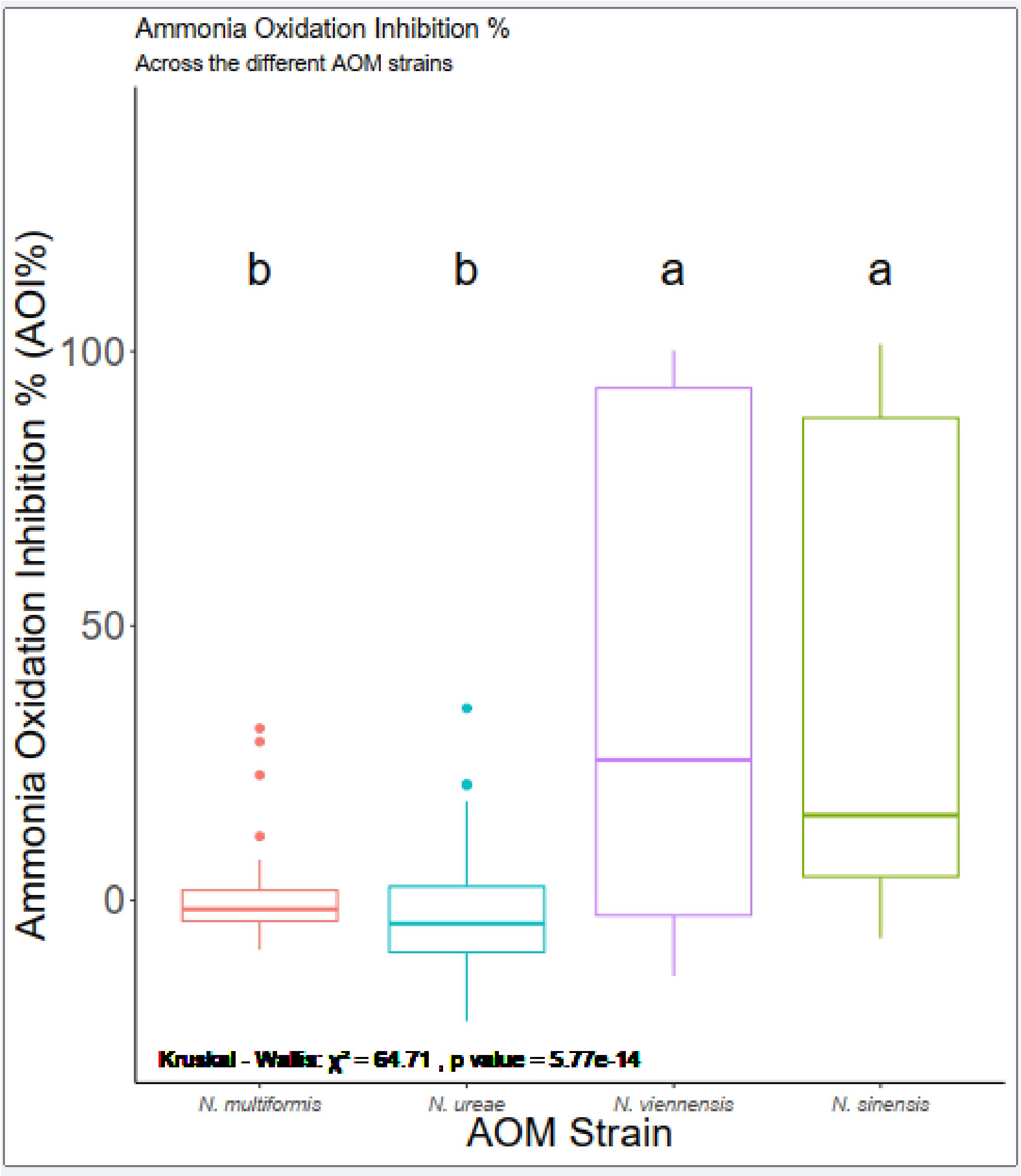

Supplementary Fig. S6 Comparison of mean ammonia oxidation inhibition (AOI, %) values for the high concentration (20 mg L^{-1}) of the tested triterpenoids on the different ammonia-oxidizing archaeal (AOA: *Nitrososphaera viennensis* and *Nitrosotalea sinensis*) and ammonia-oxidizing bacterial strains (AOB: *Nitrosospira multiformis* and *Nitrosomonas ureae*). Boxes denoted by different lowercase letters indicate statistical significance and grouping according to Kruskal – Wallis Dunn's post – hoc test (p < 0.05).
